# Property space mapping of *Pseudomonas aeruginosa* permeability to small molecules

**DOI:** 10.1038/s41598-022-12376-1

**Published:** 2022-05-17

**Authors:** Inga V. Leus, Jon W. Weeks, Vincent Bonifay, Yue Shen, Liang Yang, Connor J. Cooper, Dinesh Nath, Adam S. Duerfeldt, Jeremy C. Smith, Jerry M. Parks, Valentin V. Rybenkov, Helen I. Zgurskaya

**Affiliations:** 1 Department of Chemistry and Biochemistry, University of Oklahoma, 101 Stephenson Parkway, Norman, OK 73019 USA; 2 Graduate School of Genome Science and Technology, University of Tennessee, Knoxville, TN 37996 USA; 3 Biosciences Division, Oak Ridge National Laboratory, Oak Ridge, TN 37831 USA; 4 Department of Medicinal Chemistry, University of Minnesota, 717 Delaware St. SE, Minneapolis, MN 55414 USA; 5 Department of Biochemistry and Cellular and Molecular Biology, University of Tennessee, Knoxville, TN 37996 USA

**Keywords:** Chemical biology, Computational biology and bioinformatics, Microbiology

## Abstract

Two membrane cell envelopes act as selective permeability barriers in Gram-negative bacteria, protecting cells against antibiotics and other small molecules. Significant efforts are being directed toward understanding how small molecules permeate these barriers. In this study, we developed an approach to analyze the permeation of compounds into Gram-negative bacteria and applied it to *Pseudomonas aeruginosa*, an important human pathogen notorious for resistance to multiple antibiotics. The approach uses mass spectrometric measurements of accumulation of a library of structurally diverse compounds in four isogenic strains of *P. aeruginosa* with varied permeability barriers. We further developed a machine learning algorithm that generates a deterministic classification model with minimal synonymity between the descriptors. This model predicted good permeators into *P. aeruginosa* with an accuracy of 89% and precision above 58%. The good permeators are broadly distributed in the property space and can be mapped to six distinct regions representing diverse chemical scaffolds. We posit that this approach can be used for more detailed mapping of the property space and for rational design of compounds with high Gram-negative permeability.

## Introduction

*Pseudomonas aeruginosa* is a challenging pathogen that causes a variety of infections in humans and animals. Only a few therapeutic options are available for *P. aeruginosa* infections and those fail against multidrug-resistant isolates^1,2^. The challenge posed by this bacterium is exacerbated by a lack of success in discovering new small molecules with antibacterial activities against this pathogen.

The major reason why *P. aeruginosa* presents such a formidable therapeutic challenge is that the cells are protected by an effective drug permeability barrier composed of two lipid membranes and reinforced by active multidrug efflux pumps^3^. Currently, major efforts are being directed toward understanding and overcoming this drug permeation barrier and optimizing drug potencies against *P. aeruginosa* and other Gram-negative pathogens^4–6^.

Drug permeation studies using artificial membranes such as parallel artificial membrane permeability assays (PAMPA) or cell cultures (e.g., Caco-2 assays) are highly informative about drug oral bioavailability in mammalian hosts and are used broadly during compound optimization studies (see for example^7,8^). Recently, these approaches have been combined with traditional statistical and evolving machine learning (ML) approaches to develop predictive computational models and to identify physicochemical properties that could help with optimization of compound permeation and intracellular accumulation^9–11^. Among notable successes are Lipinski’s “rule of five” of drug solubility and permeation that is broadly used in medicinal chemistry optimization of oral bioavailability of human therapeutics^12^ and a predictive model for drug accumulation in *Caenorhabditis elegans*^13^.

In bacteria, analyses of intracellular accumulation of a broad range of chemical structures have been enabled by liquid chromatography–tandem mass spectrometry (LC–MS/MS), an approach first applied to bacteria by Cai and coworkers^14^. Later, Tan and coworkers used LC–MS/MS to analyze concentration-dependent accumulation of salicyl-AMS (adenosine monosulfamate; 5´-*O*-sulfamoyladenosine), an inhibitor of siderophore biosynthesis in *Escherichia coli*^15^. Accumulation at a 30-min timepoint was determined for ten acyl-AMS analogues in *E. coli*, *Bacillus subtilis* (Gram-positive), and *Mycobacterium smegmatis*, in the presence or absence of efflux pump inhibitors^15^. Pearson pairwise correlation coefficients were determined for accumulation under each condition versus a set of 20 calculated physicochemical parameters. This approach identified significant positive correlations between accumulation in *E. coli* and hydrophobicity.

More recently, Hergenrother and coworkers conducted a larger analysis of a diversity library of 100 compounds built around five major natural product-derived scaffolds^16,17^. Notably, they found only 12 positively charged compounds that accumulated significantly in *E. coli*, and, within this subset, no correlation was observed with hydrophobicity or molecular weight, in contrast to some early studies of radioactively labeled fluoroquinolones^18,19^. However, all 12 compounds contained amines, 8 of which were primary amines. Calculations of physicochemical descriptors, synthesis of additional analogues, and application of a random forest classification model to 68 compounds revealed that rotatable bonds and globularity were negative predictors of accumulation while the amphiphilic moment, the distance between hydrophobic and hydrophilic portions of a molecule, was a positive predictor.

Along the same lines, Erwin and coworkers evaluated the accumulation of > 100 inhibitors of NAD-dependent DNA ligase (LigA) in *E. coli* using LC–MS/MS detection^20^. They found no correlations between the size and hydrophobicity and the intracellular accumulation of these inhibitors. In agreement with early studies of small sets of compounds^21,22^, they also found poor correlation between the overall level of bacteria-associated compound and antibacterial activity in compounds with matched biochemical activities.

The varying significance of physicochemical properties such as hydrophobicity and molecular weight in the intracellular accumulation of compounds is a consequence of the complex nature of the Gram-negative permeability barrier. Active efflux pumps, which transport compounds across both the inner and the outer membranes (OMs), act synergistically with the membranes, together forming a permeability barrier for any given compound^23,24^. This synergistic relationship manifests as highly non-linear changes in intracellular accumulation levels depending on the extracellular concentration of compounds and the species-specific compositions of efflux pumps and the OM. An additional difficulty is that the above studies analyze small sample sizes with limited diversity compared to the entirety of chemical property space.

For historic reasons, most of the previous drug permeation studies have been limited to *E. coli.* However, these data are not fully applicable to all pathogens, because the cell envelope of this species is relatively permeable to small molecules. In contrast, the cell envelope of *P. aeruginosa* is notably less permeable to certain compounds than that of *E. coli*^25^ due to the presence of general porins located in the *E. coli* OM that enable diffusion of amphiphilic molecules up to 600 Da in size^26^. The OM of *P. aeruginosa* contains only substrate-specific porins that limit natural uptake to amino acids, simple sugars, and short peptides^27^. Interestingly, inactivation of all characterized and predicted porin-like proteins in *P. aeruginosa* led to only minor changes in antibiotic susceptibility, suggesting that almost all antibiotics and diverse hydrophilic nutrients can bypass porins and instead permeate directly through the OM lipid bilayer^27^. In addition, *P. aeruginosa* cells express several multidrug efflux pumps with partially overlapping substrate specificities that pump out compounds that do manage to permeate slowly across the OM^28,29^.

The contributions of the OM barrier and active efflux in antibacterial activities of compounds can be separated by comparing these activities in wild-type, efflux-deficient, hyperporinated, and ‘barrierless’ (i.e., both hyperporinated and efflux-deficient) strains^30,31^. In combination with machine learning techniques, such analyses identified physicochemical properties of compounds predictive of their interactions with proteins and lipids of the OM and active efflux pumps and showed that each pathogen presents its own unique challenges. Both barriers (i.e., OM permeability and efflux avoidance) must be considered during antibiotic development efforts, because optimizing the properties of compounds to overcome one barrier tends to result in detrimental properties against the other barrier^30,32^.

In this study, we developed an LC–MS approach to analyze the accumulation of compounds in *P. aeruginosa*. The approach avoids a washing step, the modification that enables analyses of compounds with low affinity to the cell, but also limits the approach to compounds with low non-specific binding to the filters. The insight into compound permeation is derived from measurements in four strains of *P. aeruginosa* with differing permeability barriers. We assembled a library of compounds with properties representative of drug-like libraries and analyzed the accumulation of 83 compounds in four *P. aeruginosa* strains at several extracellular concentrations and two time points (Table S1). We defined the “good” permeators as compounds that accumulate in wild type cells linearly with increasing concentration but that are not affected by compromising permeation barriers and have low non-specific retention on control filters. We further developed a machine learning algorithm that selects a descriptor set with the minimal synonymity between its members and uses this set to generate a unique classification model. The high accuracy of the model (ranging between 85 and 97%) indicates that it recapitulates the permeation properties of the compounds. Good permeators into *P. aeruginosa* were broadly variable and could be mapped to six distinct regions in chemical property space representing diverse chemical scaffolds. We posit that our strategy can be used for further, more detailed mapping of property space and for the rational design of compounds with optimized Gram-negative permeability.

## Results

### A 96-well filter assay for quantifying the contributions of efflux and the OM barrier to compound penetration into *P. aeruginosa*

The kinetics of intracellular accumulation of compounds is expected to be strongly affected by compound affinities to intracellular binding sites^23^. To test this notion, we first analyzed the accumulation of the radioactively labeled antibiotics ciprofloxacin and tetracycline, which are known to have strong affinities to intracellular targets in bacterial cells, as well as the human therapeutics oxymetazoline, nepafenac, efavirenz, delavirdine and metoprolol, which have unknown affinities to bacterial cells. As expected, measurements of minimal inhibitory concentrations (MICs) showed that ciprofloxacin and tetracycline inhibit the growth of the wild type *P. aeruginosa* PAO1 at low micromolar concentrations (Table 1). The MICs of these antibiotics improved in both the hyperporinated PAO1-Pore and the efflux-deficient PΔ3 cells, which lack the three major efflux pumps Δ*mexAB*, Δ*mexCD*, and Δ*mexXY.* However, the antibacterial activity of ciprofloxacin was mostly affected by efflux, as seen from a modest, two-fold effect of hyperporination, whereas the activity of tetracycline was affected by both the efflux and OM barriers. The “barrierless” PΔ3-Pore strain was at least 8- to 16-fold more susceptible to both antibiotics than PAO1. In contrast, no measurable antibacterial activities were observed for the other compounds for concentrations below 1 mM, except that efavirenz had an MIC of 25 mM in PΔ3-Pore cells.

**Table 1:** Minimum inhibitory concentration of antibiotics and human therapeutics in four *P. aeruginosa* strains with varying permeability barriers.

Compound	MIC, µM (mg/L)			
	PAO1	PAO1-Pore	PΔ3	PΔ3-Pore
Tetracycline	12.5 (5.56)	0.39 (0.17)	0.78 (0.35)	0.78 (0.35)
Ciprofloxacin	0.19 (0.06)	0.09 (0.03)	0.05 (0.02)	0.02 (0.01)
Metoprolol	> 1000	> 1000	> 1000	> 1000
Oxymetazoline	> 1000	> 1000	1000	1000
Nepafenac	> 1000	> 1000	> 1000	> 1000
Efavirenz	> 1000	> 1000	> 1000	25 (7.89)
Delavirdine	> 1000	> 1000	> 1000	> 1000

We next optimized conditions and analyzed time- and concentration-dependent accumulation of [^14^C]-ciprofloxacin and [^3^H]-oxymetazoline in four strains of *P. aeruginosa* with variable efflux and OM permeability barriers using a 96-well filter plate assay. We found that washing of filtered cells with the incubation medium differentially affected the retention of [^14^C]-ciprofloxacin and [^3^H]-oxymetazoline (Fig. S1), suggesting that compound variability in affinities to intracellular binding sites could strongly affect the outcomes of the assay. Therefore, washing of filtered cells was omitted and filters were removed from plates to reduce non-specific background (see Methods). As described in Methods, under the conditions of the assay, each filter retained 4.3 ± 0.7 µL of solution, and cells displaced 2.8 ± 0.2 µL of this volume (67%) (Fig. S1). Thus, the assay is expected to report accurate values for compounds that accumulate in cells but will not be informative for compounds unable to accumulate in at least one of the four strains.

**Figure 1:**
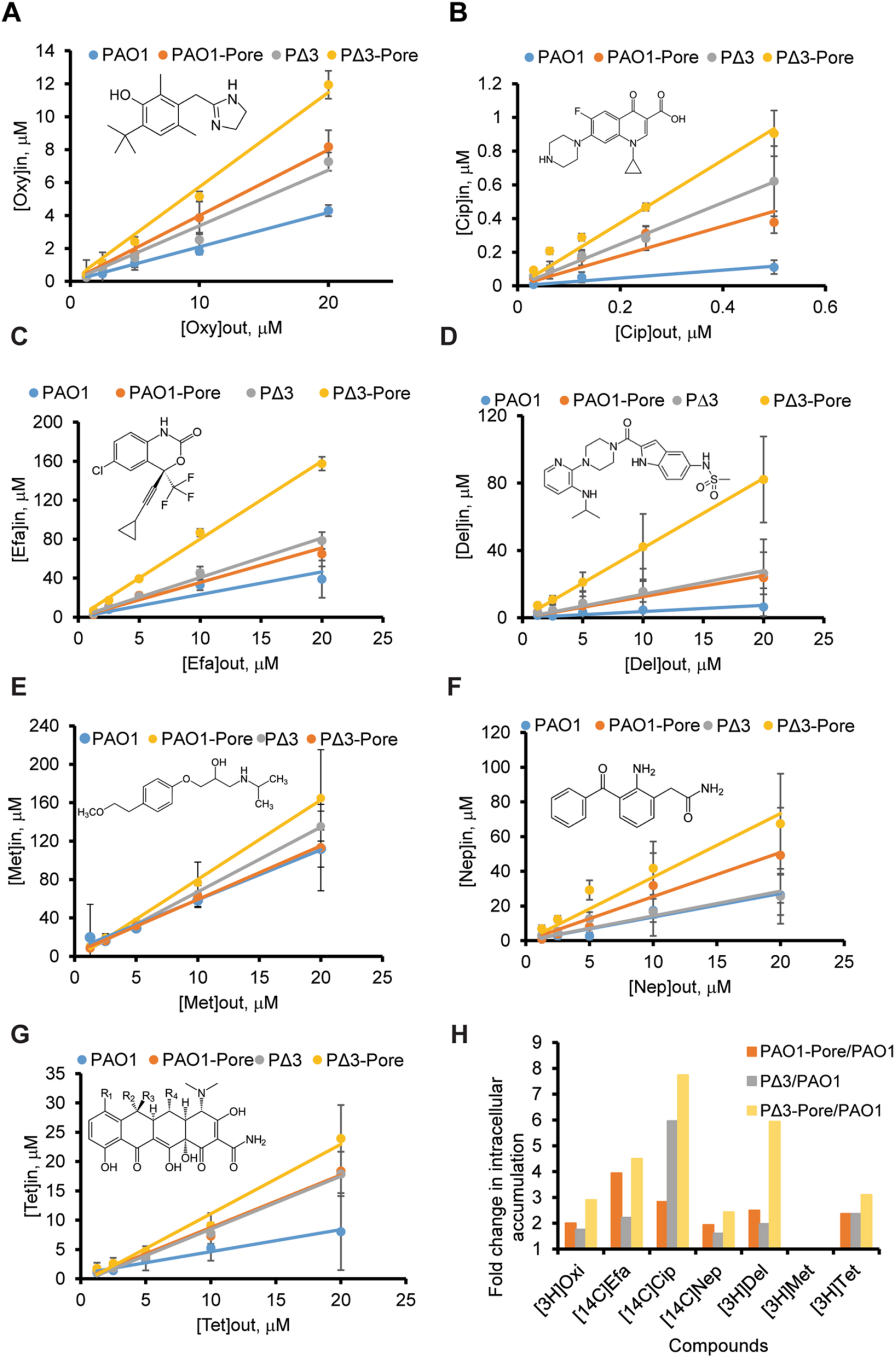
Intracellular accumulation profiles of radioactively labeled compounds in four strains of *P. aeruginosa* after 20 min of incubation: (**A**) [^3^H]-oxymetazoline (Oxy); (**B**) [^14^C]-ciprofloxacin (Cip); (**C**) [^14^C]-efavirenz (Efa); (**D**) [^3^H]-delavirdine (Del); (**E**) [3H]-metaprolol (Met); (**F**) [^3^H]-nepafenac (Nep); **G**. [^3^H]-tetracycline (Tet). Extracellular (out) and intracellular (in) concentrations were calculated based on specific radioactivities of compounds. (**H**) Fold change (represented as slope ratios) in the intracellular accumulation of compounds in hyperporinated, efflux-deficient and “barrierless” cells in comparison to the wild type PAO1.

To test the validity of the assay, we next measured the accumulation of [^14^C]-ciprofloxacin and [^3^H]-oxymetazoline into otherwise isogenic strains of *P. aeruginosa* that differ in the permeability of their cell envelope. Since the composition of cell membranes does not change significantly with hyperporination and efflux inactivation^29^, we expect that non-specific binding remains the same for different strains and the differences in the accumulation levels between the strains reflect the differences in the intracellular accumulation. Kinetic experiments showed that both [^14^C]-ciprofloxacin and [^3^H]-oxymetazoline reached steady-state levels within the first minutes of incubation (Figs. S2, S3). The steady-state levels increased linearly with increasing concentrations of the compounds (Fig. 1A,B). Furthermore, these accumulation levels varied between the strains and were higher in the efflux-deficient and hyperporinated cells than in the wild type PAO1. Efflux inactivation or hyperporination resulted in a similar increase in the accumulation of [^3^H]-oxymetazoline, and the levels were highest in PΔ3-Pore cells. In contrast, inactivation of efflux led to significantly higher levels of accumulation of [^14^C]-ciprofloxacin than did hyperporination but again the levels were highest in PΔ3-Pore cells. This major contribution of efflux in the intracellular accumulation of ciprofloxacin was also observed in its MICs in different *P. aeruginosa* strains (Table 1).

The above findings show that the developed assay allows analyses of the kinetics of drug accumulation in cells and can distinguish between the contributions of active efflux and the OM barrier. Importantly, in the time-course of the experiment, changes in the intracellular levels in wild type PAO1 were very small for both radioactive compounds, suggesting that the compounds permeate these cells very slowly, even at the highest concentrations considered. In addition, at the highest concentration of [^3^H]-oxymetazoline (20 µM), the signal declined during the first 5 min of incubation in PAO1 cells. This result suggests that when compounds do not accumulate in cells, they can be lost due to non-specific binding to plates or aggregation.

Accumulation of other radioactively labeled compounds was also concentration-dependent and varied between the four *P. aeruginosa* strains (Fig. 1). Hyperporination had the strongest effect on the accumulation of [^14^C]-efavirenz, [^3^H]-nepafenac and [^3^H]-delavirdine, whereas the “barrierless” PΔ3-Pore cells accumulated the highest levels of compounds (Fig. 1C,F). The accumulation of delavirdine and tetracycline was equally affected by inactivation of efflux and hyperporination (Fig. 1D,G), whereas metaprolol accumulation was not affected by either of the two barriers (Fig. 1E). When intracellular accumulation of compounds was normalized to that of the PAO1 cells, the ratio of concentration slopes even for PΔ3-Pore varied between one- and eightfold (Fig. 1H). Except for ciprofloxacin, inactivation of efflux alone and hyperporination of cells alone generated modest slope ratios of 1.5–3. Thus, the assay is sensitive to differences in permeation and can be used with structurally diverse compounds.

### Properties of the focused library of compounds for analyses

Previous studies have shown that the physicochemical space of antibiotics is broader than other drugs and includes more hydrophilic compounds, such as beta-lactams, which penetrate the cells through water-filled OM porins, as well as some large compounds, such as vancomycin, which act on cell surfaces and do not need to penetrate the cell envelope^33^. In addition, the presence of primary amines and positive charges are in general associated with increased penetration into some bacterial cells^34^. We next analyzed a focused library of 12,000 commercially available compounds and selected 220 compounds with PSA 50 + , MW < 2000 Da, and cLogD_7.4_ < 5 for further analyses.

Approximately 10% of the 220 purchased compounds possessed antibacterial activities in at least one of the four *P. aeruginosa* strains grown in MOPS-M9 medium and Luria–Bertani (LB) broth and these included representatives of fluoroquinolones, sulfonamides, cyclines and linezolid (Tables 2 and S2). After elimination of insoluble compounds and optimization of the LC–MS method, 98 compounds were selected for further analyses, including six synthesized trisubstituted piperazin-2-one derivatives (Fig. 7), out of which the intracellular accumulation of 83 compounds was measured and for 66 compounds it was quantified (see below, Table S1).

**Table 2:** MICs of antibiotics analyzed by LC–MS in four strains of *P. aeruginosa* grown in MOPS-M9 medium*.*

Antibiotic	MICs, μM (mg/L)			
	PAO1	PAO1-Pore	P∆3	P∆3-Pore
Doxorubicin	200 (108.7)	12.5 (6.8)	25 (13.6)	3.2 (1.7)
Levofloxacin	0.5 (0.18)	0.031 (0.011)	0.25 (0.2)	0.008 (0.003)
Norfloxacin	1.25 (0.4)	0.2 (0.06)	0.625 (0.2)	0.1 (0.03)
Sulfaphenazole	> 200 (> 62.9)	12.5 (3.9)	200 (62.9)	3.2 (1.0)
Sulfameter	> 200 (> 56.1)	12.5 (3.5)	25 (7.0)	6.25 (1.75)
Sulfamethazine	> 200 (> 60.1)	50 (15.0)	100 (30.0)	12.5 (3.75)
Sulfathiazole	50 (12.8)	3.2 (0.8)	6.25 (1.6)	1.6 (0.4)
Sulfisoxazole	> 200 (> 53.7)	> 200	> 200	> 200
Sulfadimethoxine	> 200 (62.1)	25 (7.8)	200 (62.1)	1.6 (0.5)
Prulifloxacin	0.5 (0.23)	0.016 (0.01)	0.125 (0.06)	0.004 (0.002)
Nadifloxacin	5 (1.8)	0.0625 (0.02)	0.625 (0.23)	0.0156 (0.01)
Pazufloxacin mesylate	0.5 (0.21)	0.0625 (0.03)	0.125 (0.05)	0.031 (0.01)
Moxifloxacin hydrochloride	2.5 (1.1)	0.0625 (0.03)	0.625 (0.27)	0.0313 (0.01)
Difloxacin hydrochloride	2.5 (1.1)	0.0625 (0.03)	0.313 (0.14)	0.0313 (0.01)
Gatifloxacin	2 (0.8)	0.0625 (0.02)	0.5 (0.2)	0.031 (0.01)
Oxolinic acid	20 (5.2)	0.625 (0.16)	2.5 (0.65)	0.078 (0.02)
Enrofloxacin	2 (0.7)	0.0625 (0.02)	0.5 (0.18)	0.016 (0.01)
Pefloxacine mesylate	2 (0.9)	0.25 (0.11)	0.5 (0.21)	0.0625 (0.03)
Sparfloxacin	2 (0.8)	0.031 (0.01)	0.5 (0.2)	0.016 (0.01)
Lomefloxacin	2.5	0.125	0.625	0.125
Ciprofloxacin	1 (0.33)	0.0625 (0.02)	0.25 (0.08)	0.031 (0.01)
Linezolid	> 200 (> 67.5)	200	200	25

**Figure 2:**
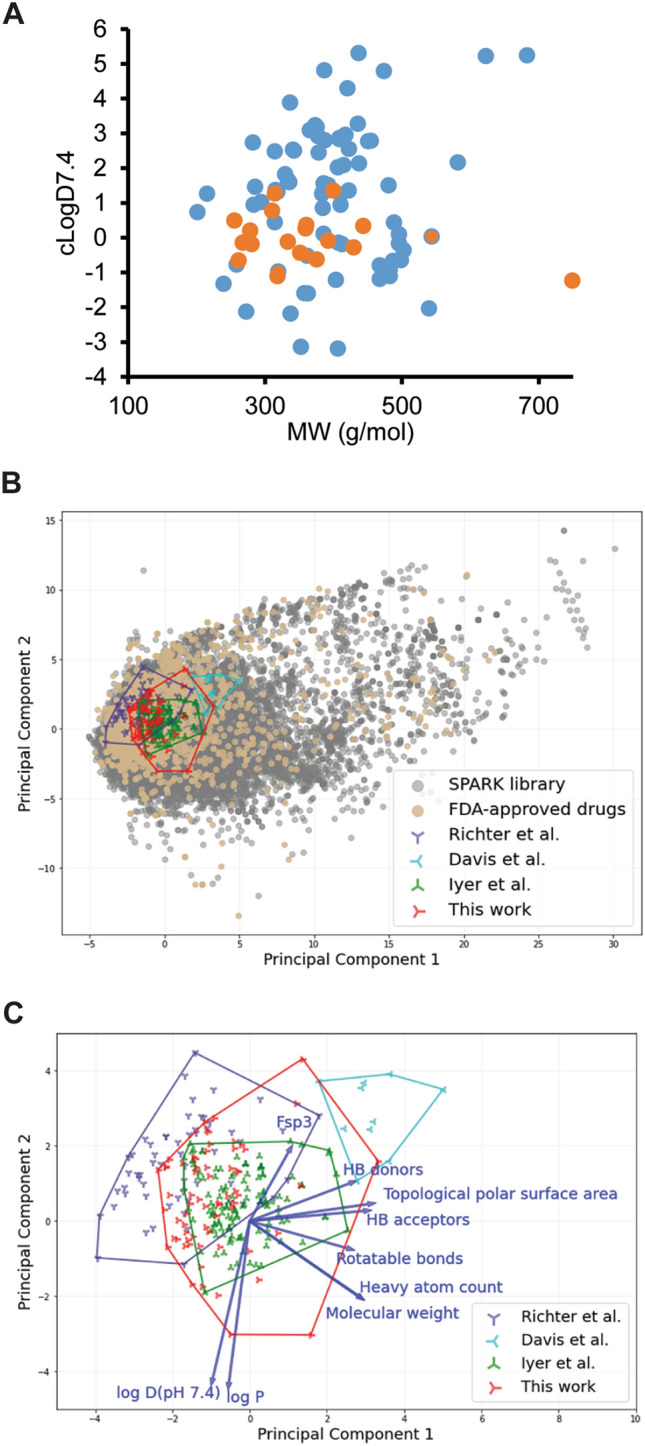
Properties of the library of 66 compounds analyzed in this study. (**A**) Size and lipophilicity of the analyzed compounds as represented by MW and cLogD_7.4_ descriptors. Antibiotics are highlighted in orange. (**B**) Principal component analysis of diversity in the chemical space of molecules from SPARK library^35^, Richter et al.^16^, Davis et al.^15^, Iyer et al.^20^, FDA- approved drugs (extracted from^36^) and this study. The outermost data points from the four studies are linked to show the relative size of the sampled space. (**C**) Zoom-in view of (**B**) and the loading vectors for this plot. Data points for the SPARK library and FDA-approved drugs are hidden for clarity. Molecular weight, hydrogen bond donors and acceptors, topological polar surface area, rotatable bounds and heavy atom count are the major contributing factors to PC1, whereas LogP, logD and the fraction of sp^3^ hybridized carbon atoms (Fsp^3^) are major contributors to PC2.

The analyzed 66 compounds vary in molecular weight (MW) between 200 and 750 Da and have cLogD_7.4_ values between 5.0 and -3.5 (Fig. 2A). We next calculated nine physico-chemical properties including the molecular weight, logP, the number of hydrogen bond donors and acceptors, clogD_7.4_, the topological polar surface area, the fraction of sp^3^ hybridized carbon atoms (Fsp3), the heavy atom count, and the number of rotatable bonds for the analyzed compounds. These properties were also calculated for the SPARK compound library^35^, FDA-approved drugs^36^, and compounds whose accumulation in *E. coli* was analyzed in previous studies^15,20,34^. Principal component analysis showed that the chemical space covered in this work is broader than the space covered by previous studies^15,20,34^ (Fig. 2B-C)*.* The distribution of properties of this library surrounds that of Iyer et al.^20^ in every feature (Fig. S4) and that of Richter et al.^34^ in principal component 1 (PC1), which is represented by molecular weight, number of hydrogen bond donors and acceptors, topological polar surface area, rotatable bounds, and heavy atom count (Fig. 2C and S4). The distribution of the compounds analyzed by Richter et al.^34^ is wider in the Fsp3 property space but narrower in logP and logD, resulting in a slightly narrower distribution in principal component 2 (PC2). The partial overlap in PC1 between our compounds and the Richter et al. set is due to an offset in the range of values for the topological polar surface area and the number of hydrogen bond acceptors. For example, the number of hydrogen bond acceptors ranges from 0–8 in the Richter et al. set and from 2–12 in the present study.

### Quantification of the effect of efflux and the OM barrier in compound penetration into *P. aeruginosa*

The 96-well filter assay described above was next used for LC–MS-based quantification of compound penetration into *P. aeruginosa* cells. To reduce variability, non-physiological responses, and matrix effects, cells were grown in the optimized M9 minimal medium (see Methods) and all subsequent steps were carried out at room temperature.

**Figure 3:**
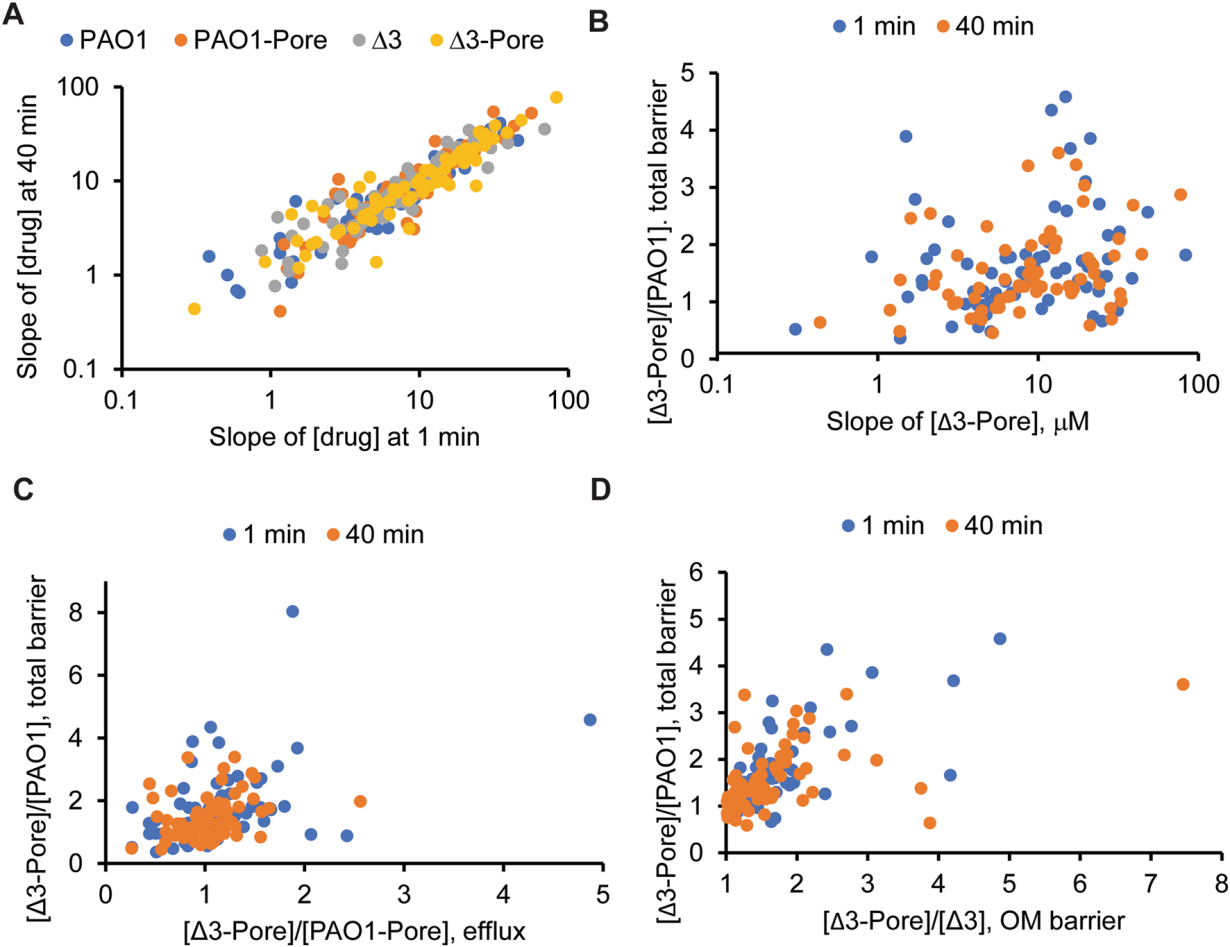
Intracellular accumulation of the analyzed library of 66 compounds. (**A**) Slopes of intracellular accumulation of compounds at 1 min and 40 min incubation times. (**B**) Plot of the slopes of measured intracellular accumulation in the “barrierless” strain as a function of the fold difference in accumulation in PAO1 and “barrierless” strain. (**C**) and (**D**) Comparison of fold change in the compound accumulation for the PΔ3-Pore/PAO1 (Total barrier) and PΔ3-Pore/PAO1-Pore (Efflux) or PΔ3-Pore/PΔ3 (OM).

Compound accumulation was analyzed at four concentrations: 10, 20, 40 and 80 µM. Kinetic analyses of the radioactively labeled compounds described above showed that compounds vary in the time needed to reach steady-state levels, with some compounds reaching the steady state in all four strains within the first 1–2 min of incubation and others requiring longer incubation times, especially in the case of PAO1 cells (Fig. 3). In contrast, for some compounds longer incubation times led to a decrease in compound concentration (Fig. S2). Therefore, we chose to analyze the compound library accumulation in cells incubated with compounds for 1 min and 40 min.

The LC–MS method developed here enabled concentration-dependent quantification of 83 compounds including 22 antibiotics in four strains of *P. aeruginosa.* After subtraction of signals from the excluded volume of empty filters, the positive concentration values were obtained for 66 compounds, which were further analyzed (Table S1). For most of the compounds, the calculated concentration slopes of compound accumulation in four strains at 1 and 40 min correlated well with each other (Fig. 3A). At each concentration, the accumulation levels varied by orders of magnitude depending on the specific compound. However, with few exceptions, the differences between the strains were relatively small. Overall, we found that the accumulation levels in the “barrierless” PΔ3-Pore cells at 1 and 40 min trended positively with the ratios of concentration slopes obtained in PΔ3-Pore and PAO1 cells (Fig. 3B). This result suggests that for certain compounds the high accumulation levels in PΔ3-Pore are due to the removal of the permeation barriers.

To normalize for differences in the affinities of compounds for intracellular targets, we also used: (i) the efflux ratios expressed as the ratios of measured concentrations or concentration slopes in efflux-deficient and efflux-proficient cells with the hyperporinated OM (PΔ3-Pore/PAO1-Pore), (ii) the OM ratios expressed as the respective ratios in efflux-deficient cells with normal and hyperporinated OMs (PΔ3-Pore/PΔ3), and (iii) the total barrier (PΔ3-Pore/PAO1). No significant correlation was found between the efflux and the total barrier ratios, suggesting that the contribution of efflux varied broadly between compounds, and was not the major contributor to the intracellular accumulation (Fig. 3C). In contrast, the OM ratios clearly correlated with the total barrier (Fig. 3D). This result suggests that the permeation across the OM is the dominating factor for intracellular accumulation of the analyzed compounds.

### Classification of compounds

The measured levels of compound accumulation were analyzed using machine learning to determine which features of the compounds are most important for enabling their cellular accumulation and to determine their optimal values. For modeling, we used compound concentration slopes of the accumulation levels observed after 1 min (A1) and 40 min (A2) incubations, which were approximated as straight lines with y-intercept set to zero. To accentuate the permeability aspect, the accumulation slopes in efflux-deficient and hyperporinated strains were normalized to those in the parental PAO1 strain. In several cases, the slopes were lower in the permeabilized cells than in wild type cells, presumably due to data scatter or low signal (Table S1). Such changes cannot be attributed to the porination state or efflux deficiency of the cell. Therefore, we attributed such cases to the lack of an increase in permeability and set those ratios equal to 1.

**Figure 4:**
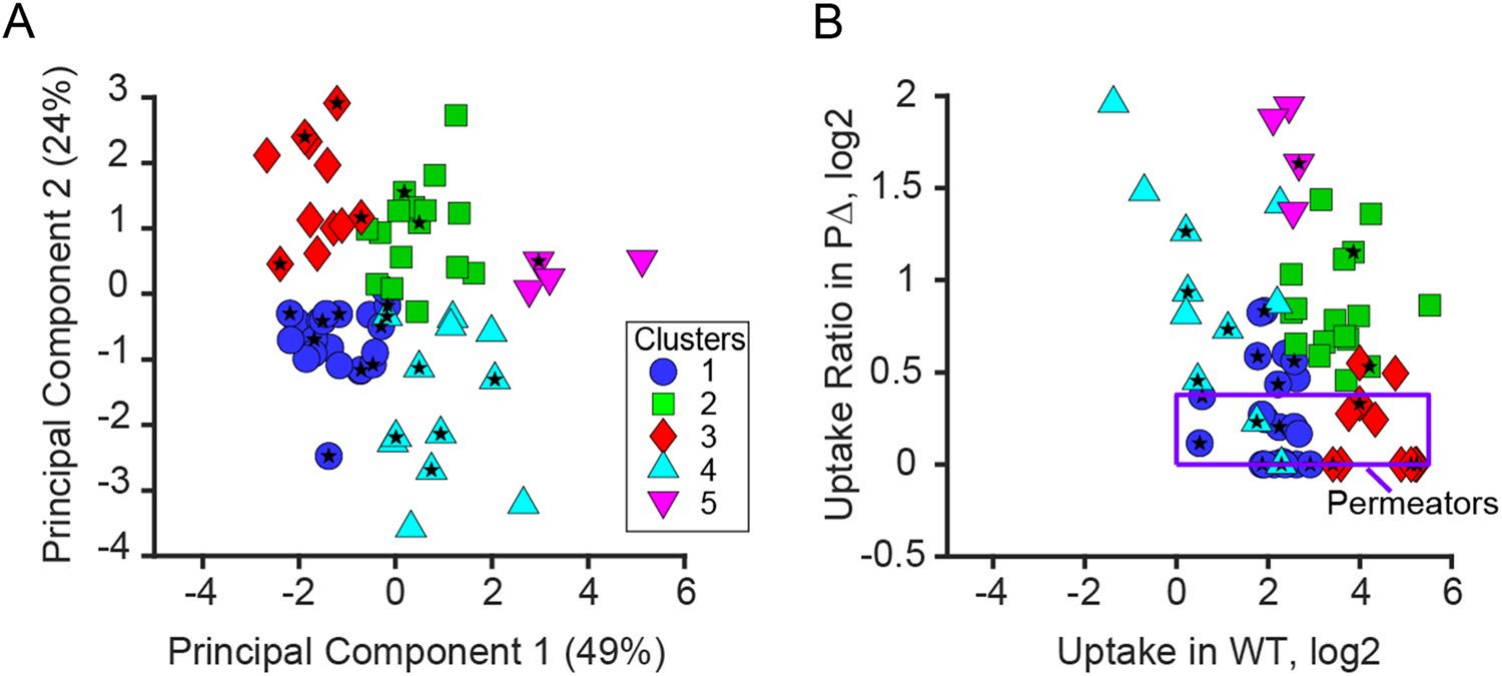
Distribution of uptake data in the activity space in PC (**A**) and native (**B**) coordinates. Crosses mark centers of clusters determined using *k*-means clustering. Violet rectangle encompasses compounds defined as good permeators. Stars mark antibiotics.

To help visualize the data, we performed a principal component (PC) decomposition of the data. The first two principal components were split almost equally between efflux and hyperporination ratios (PC 1) or dominated by the absolute levels of compound accumulation (PC 2; Table S3). Elbow analysis of the unexplained variance suggested the existence of five clusters, which were well separated in the principal component view (Fig. 4A) and in native coordinates (Fig. 4B). All clusters comprised compounds that are affected to a various extent by *P. aeruginosa* permeability barriers. To formalize further analysis, we defined as the target subgroup those compounds that (i) accumulate at or above extracellular levels, (ii) exhibit relatively low signal on empty filters; and (iii) whose uptake does not increase by more than 30% by hyperporination combined with efflux inactivation (boxed in Fig. 4B). A total of 25 compounds satisfied these criteria and were classified as good permeators. Most of the good permeators were found in clusters 1 and 3.

### Random Forest classification

**Figure 5:**
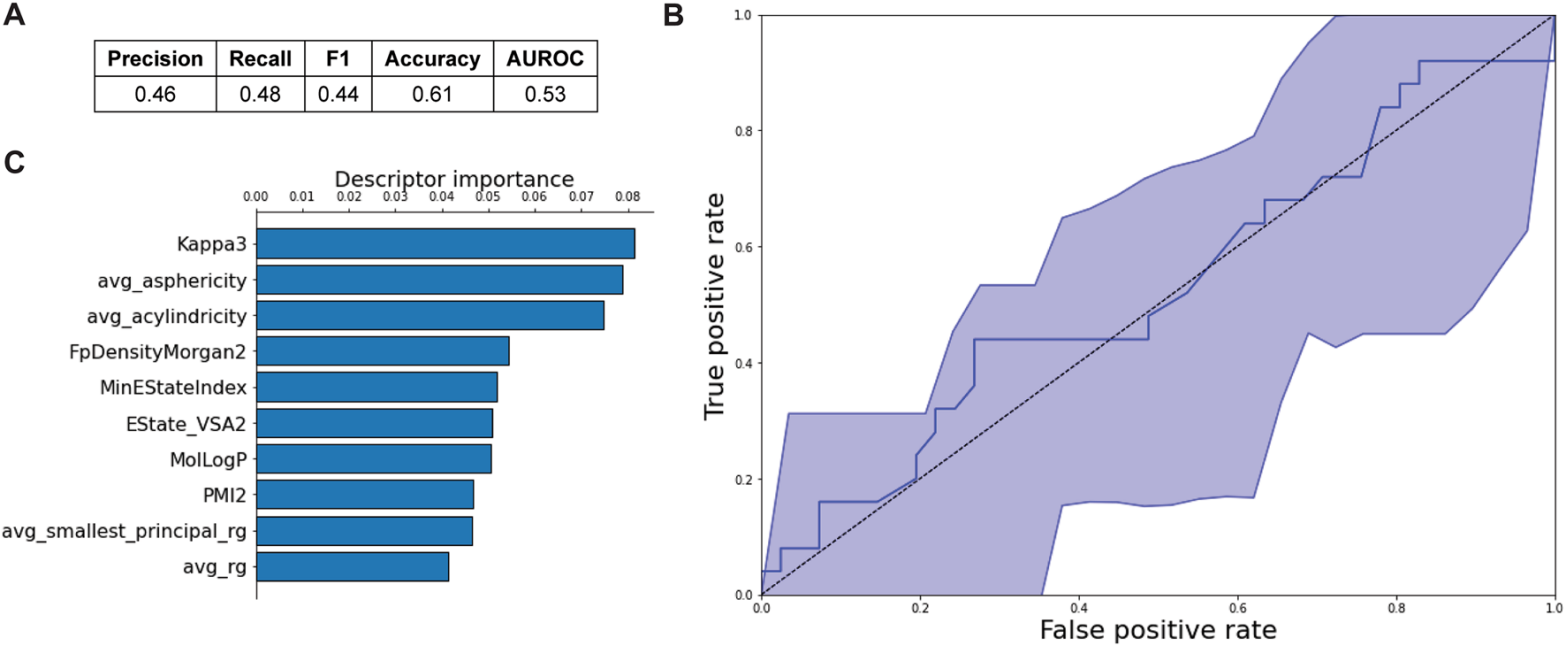
Random forest model performance metrics and diagrams. (**A**) Performance metrics, (**B**) ROC curve derived from leave-one-out cross validation with 95% confidence bands, (**C**) Importance bar plot of top ten features.

The dataset of 66 compounds is relatively small, but it is comparable to previous studies^16,20^. We first trained a random forest binary classification model using the above LC-MS data and 27 2D and 3D physicochemical descriptors for each compound that were pre-selected from a larger descriptor pool by clustering analysis (see Methods). The model was designed to distinguish between permeators and non-permeators as defined from the principal component analysis (Fig. 4B). The model achieved only 61% accuracy, 48% recall and 46% precision when tested on leave-one-out cross-validation (Fig. 5A). The area under the receiver-operator characteristic (AUROC) curve was only 0.53. The broad confidence interval (Fig. 5B) indicates that the performance of the model is largely dictated by which compounds are included in the training set for a given cross-validation step. This behavior implies that a larger compound set would likely significantly improve the precision of the model. Descriptors derived from molecular dynamics (MD) simulations are prevalent among the top ten, suggesting that molecular shape, conformational flexibility, and dynamics are important for distinguishing permeators from non-permeators (Fig. 5C). Examples include shape-based descriptors such as the Hall-Kier kappa3 molecular shape descriptor, the average acylindricity and asphericity, i.e., the deviation from cylindrical and spherical symmetries, and the radius of gyration (average R_g_ and average smallest principal R_g_). Other top descriptors associated with favorable uptake include lipophilicity (MolLogP) and properties related to molecule topology (second principal moment of inertia, minimum absolute E-state index, and Morgan fingerprint density).

### Gnomic classification tree

One of the limitations of the RF analysis is its propensity to use all provided descriptors without any regard for potential overlap between them. To address this issue, we developed a classification algorithm, Gnomic, that helps reduce the number of synonymous descriptors in the constructed classification model and ultimately constructs a single, unique classification tree. The descriptors identified during the construction of the tree define a property space that can be used for mapping compounds with diverse activities.

**Figure 6:**
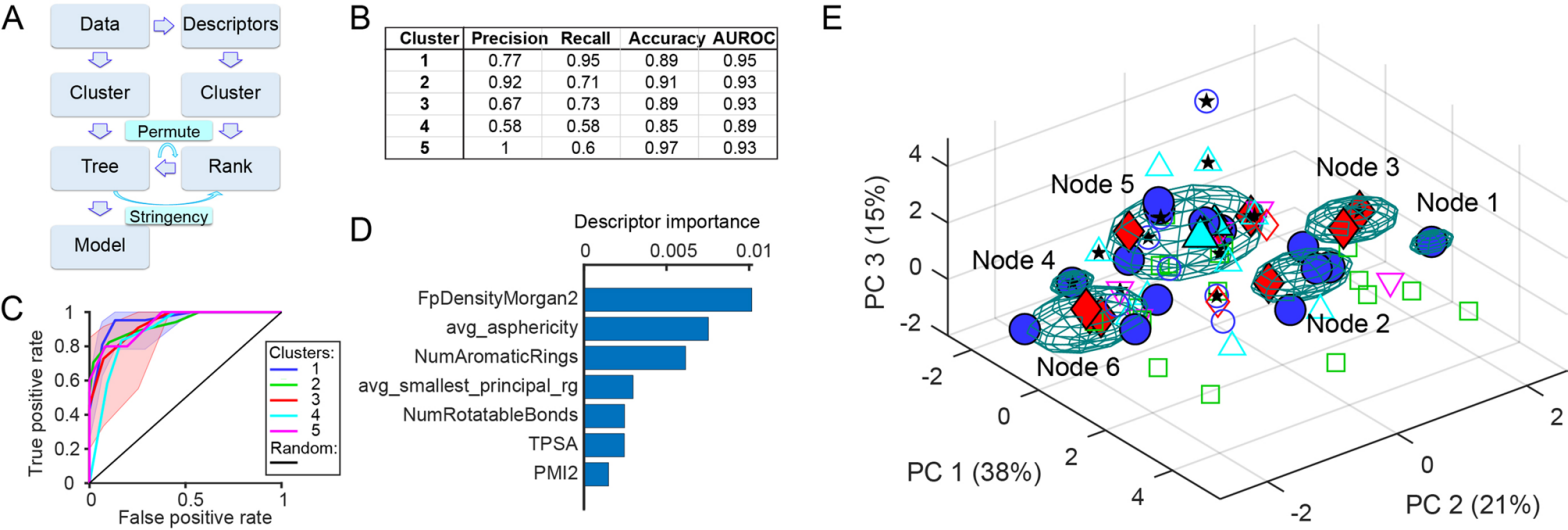
Gnomic model of compound accumulation in *P. aeruginosa*. (**A**) Gnomic algorithm. (**B**) Performance metrics for the prediction of each cluster. (**C**) Receiver operating curve of the classification model. Shaded area marks the confidence bands for cluster 2. (**D**) Descriptors selected by training the model. (**E**) Distribution of the clusters in the property space. Color coding the same as in Fig. 4.

The algorithm employs several rounds of descriptor selection (Fig. 6A). First, the descriptors are clustered as described in Methods, and a single representative descriptor is selected from each cluster. These representatives are then ranked in the order of their predictability by the other descriptors, using a regression tree, in an iterative procedure that sequentially removes the most predictable descriptor from the list. In this procedure, the misclassification error of the regression tree defines the extent of the information overlap between the tested descriptors, i.e., the smaller the error, the larger the synonymity. The misclassification error also offers a natural measure of the stringency of the procedure. As a result, the synonymous descriptors are removed from the set prior to modeling, and the stringency of the filtering can be varied simply by changing the selection threshold. Thereafter, a classification tree can be trained on the uptake data at various levels of stringency, thereby finalizing the list of descriptors. To reduce the bias caused by the initial ranking of the descriptors, the order of descriptors was randomly permuted 100 times during the construction of the classification tree at each stringency level.

Using Gnomic, we constructed models that predict the five clusters defined by the permeability of the compounds (Fig. 6). We then selected the model that yields the highest overall precision, 79%. The accuracy of the models varied between 85 and 97% and recall rates between 58% for cluster 4 and 95% for cluster 1 (Fig. 6B, C). The model operated with 7 descriptors, including those reporting on the shape of the molecule, charge and its distribution, topology of the compound, number of rotatable bonds and the presence of aromatic rings (Fig. 6D).

We next visualized the model by plotting the data in the descriptor space (Fig. 6E). Despite the high precision of the model, there was no clear set of descriptor values that defined any of the clusters. Data points from most clusters covered a sprawling, irregularly shaped chemical space that spread throughout the entire sampled region. In contrast, the separation between the clusters was much smaller than their reach.

We next mapped the subset of good permeators defined in Fig. 4B to the property space. The 25 compounds found in this set formed six clusters, one of which included only a single compound (Fig. 6E). Hereafter, we refer to these clusters in the property space as nodes to distinguish them from the clusters in the activity space defined in Fig. 4. A single cluster in the permeability space usually transformed into multiple nodes when viewed in the property space (Fig. 6E) and vice versa. The nodes comprising multiple compounds were mostly devoid of non-permeating molecules, indicating that the constructed map represents at least a fraction of the space attainable to good permeators.

Notably, some known antibiotics were found within or in the vicinity of some, but not all identified regions. Node 5 comprises six fluoroquinolones and linezolid, whereas another fluoroquinolone prulifloxacin is in node 3. Node 4 is sulfamethazine. For these antibiotics, the efflux (PAO1-Pore/PΔ3-Pore) and the OM (PΔ3/PΔ3-Pore) MIC ratios varied broadly (Table 2). This result agrees with previous studies that showed a lack of simple correlations between permeation and the antibacterial activities of antibiotics^20,23,24^. Compounds from the other nodes have not been previously associated with efficient permeation into bacteria. Thus, this study identifies new property regions in which high permeability into *P. aeruginosa* is expected. The optimal values of the descriptors that define these regions are listed in Table 3.

## Discussion

In this study, we have developed an approach to analyze the permeation of small molecules into Gram-negative bacteria and to identify physicochemical properties associated with the trans-envelope permeation. We applied this approach to a library of 66 structurally diverse compounds and identified “good” permeators and their properties. The experimental approach is based on LC–MS quantification of compound concentrations, a technique used previously to measure the intracellular accumulation of antibiotics and series of compounds with antibacterial activities^14–16,20,37^. A critical bottleneck in studies of intracellular accumulation is the separation of molecules located inside the cells from external compounds. To achieve such separation, the majority of previously reported approaches used either centrifugation through silicon oils followed by freezing and cutting cell pellets^16,20^, or vacuum filtration onto low-binding filters followed by washing the filters with an ice-chilled buffer or medium^37–39^. Depending on the approach, these separation steps are completed within several minutes and could strongly affect results for compounds that lack high affinities to extra- or intra-cellular components^23,40^ and equilibrate rapidly across membranes (Figure S1). We evaluated the approach that avoids a washing step and found it informative for analyses of compounds with and without high affinity intracellular sites.

Another important question in such measurements is how to separate “good” permeators from “bad”. The different levels of accumulation in cells with native versus disrupted permeability barriers identify compounds that are affected by specific barriers (Fig. 1) but do not necessarily distinguish between “good” and “bad” permeators into wild type cells. The absolute measured concentrations may be misleading because of non-specific binding to filters or cell debris, even for the high-affinity intracellular binders and methods with washing steps, as well as leakage of compounds from “barrierless” cells. In the presented approach, there is a simple intrinsic control for “bad” permeators: such compounds are identified from their increased intracellular accumulation in hyperporinated and efflux-deficient cells. However, careful measurements and subtraction of non-specific signal was required to identify “good” permeators. We identified compounds with strong non-specific binding to empty filters and such compounds were excluded from further analyses. We further relied on time- and concentration-dependent changes in compound concentrations and defined the “good” permeators as compounds that accumulate in PAO1 cells linearly with increasing concentration but that are not affected by compromising permeation barriers and have low non-specific retention on control filters.

The molecular signature of good permeators consisted of a mixture of distinct properties including the size and shape of the molecule, polarity, topology, and a handful of molecular fingerprints such as the number of aromatic rings or rotatable bonds (Fig. 6). These properties are often found in this type of analysis and can be readily traced to the mechanism of small molecule penetration into bacteria^15,16,30^. The true challenge was to find numeric values of these parameters that are conducive to penetration.

To solve this problem, we performed two types of analysis. First, we trained an RF model to recognize good permeators. However, due to the limited size and broad coverage of chemical space in the dataset, the performance metrics for the RF model were unsatisfactory (Fig. 5). Thus, a second machine learning approach was designed to address this problem. We developed the Gnomic algorithm (Fig. 6A), which ranks descriptors according to their unpredictability and thereby helps select descriptors with minimal information overlap. We reasoned that this approach would create an acceptable compromise between absorbing unique molecular signatures available in diverse descriptors and avoiding the synonymity between them. Clearly, the use of synonymous descriptors in a model only expands the dimensionality of the solution without improving its predictive power.

Traditionally, ML methods address this issue through the use of curvature tests, which eliminate highly correlated descriptors. The descriptor clustering step builds on this idea and sets an adaptive threshold for the acceptable correlation. However, these steps capture only pairwise correlations between descriptors and often overlook the instances when the information contributed by one descriptor is fully contained in a group of others. Gnomic searches for such instances by building regression models that predict a given descriptor through the rest. A low misclassification error in such models signifies a low informational contribution from said descriptor. This approach can be viewed as an ML equivalent of the linear algebra concept of a linear independence between vectors. Unlike in linear algebra, the predictability can recapture instances of non-linear interaction between descriptors. This feature makes it better suited for the analysis of seemingly stochastic distributions such as the one examined here.

The RF model used all 27 descriptors. In contrast, the Gnomic model identified seven descriptors with non-zero importance (Table S4). These seven descriptors comprise a reference set in the property space for inspecting compounds. The top descriptors of good permeators mainly define topological, constitutional, and molecular properties of compounds (Table S4). Among these, shape (avg_asphericity) and number of rotatable bonds (NumRotatableBonds) were highlighted in previously developed models based on the studies of either intracellular accumulation or antibacterial activities^15,16,30^. However, a closer inspection shows that the numerical values of these descriptors vary broadly between different permeation nodes and do not clearly distinguish them (Table 3).

**Table 3:** Values of top descriptors in the center of permeation nodes and their comparison to antibiotics.

Descriptor^a^	Node						AB
	1	2	3	4	5	6	
FpDensityMorgan2	1.5 ± 0	1.8 ± 0.1	2.0 ± 0.0	1.6 ± 0	2.1 ± 0.2	1.9 ± 0.1	2.0 ± 0.2
avg_asphericity	0.52 ± 0	0.40 ± 0.03	0.39 ± 0.00	0.14 ± 0	0.29 ± 0.04	0.22 ± 0.02	0.31 ± 0.06
NumAromaticRings	2.0 ± 0	2.4 ± 0.5	3.0 ± 0.0	2.0 ± 0	1.8 ± 0.4	2.0 ± 0.9	2.0 ± 0.5
avg_smallest_principal_rg	0.22 ± 0	0.23 ± 0.02	0.24 ± 0.02	0.24 ± 0	0.22 ± 0.01	0.29 ± 0.01	0.23 ± 0.03
NumRotatableBonds	4.0 ± 0	3.8 ± 0.4	4.0 ± 0.0	3.0 ± 0	3.1 ± 1.3	6.4 ± 1.0	3.7 ± 1.8
TPSA	93 ± 0	66 ± 8	116 ± 3	98 ± 0	85 ± 14	61 ± 10	98 ± 32
PMI2, × 1000	10.8 ± 0	6.9 ± 1.1	10.7 ± 0.9	1.9 ± 0	3.5 ± 1.1	3.9 ± 1.2	4.5 ± 2.5

^a^See Table S3 for definitions. AB-antibiotics.

The 25 good permeators were broadly distributed in the property space with at least six nodes in diverse scaffolds (Fig. 6E). This distribution suggests that the region occupied by good permeators is not truly fractal but has a continuous fabric at its base. We posit that other compounds that map to these nodes would penetrate *P. aeruginosa.* Moreover, other permeability nodes should be discoverable by further testing. Overall, this approach suggests a framework for mapping compound permeabilities in the chemical space and the development of predictive models for antibiotic design based on them.

The near fractal character of the permeator region explains prior difficulties in the construction of linear regression models of permeation or the discovery of a universal chemical modification that would permeabilize the molecule. Such modifications ought to be scaffold dependent. On the bright side, good permeators can be found in the vicinity of many scaffolds. Some of these scaffolds are already well known. For example, nodes 3, 4 and 5 contain antibiotics. Importantly, antibiotics from the same class, e.g., the fluoroquinolones norfloxacin and prulifloxacin can be found in several nodes because they vary significantly in their structures and properties (Tables 3 and S1). The 2-oxo-1,3-dioxol substitution on the piperazine ring and thiazeto modification on the quinoline ring of prulifloxacin affect size, shape, charge and other properties that are important for permeation (Table 3). Among good permeators are also human therapeutics, e.g., ergotamine and sitagliptin. Both drugs are oral and hence, display good epithelial permeability, and these chemotypes may contain useful structural motifs and/or connectivity patterns that can be leveraged to enhance Gram-negative permeation of other scaffolds.

The inspection of structures of good permeators did not yield a single functional group consistently present in all compounds. However, some of the moieties such as trifluoro- or diaminomethyl- substitutions are known to improve permeation across bacterial membranes^31,41^. Our results also show that the contribution of such chemical modifications to permeation across *P. aeruginosa* and other Gram-negative cell envelopes will be scaffold dependent. Further analyses of intracellular permeation and modeling of larger chemical libraries around several biologically active scaffolds using hit expansion techniques will lead to a more detailed mapping of the space of good permeators and could potentially reveal scaffold-dependent heuristics.

Optimization of antibiotic activities against Gram-negative bacteria could be strongly facilitated if the effect of a given chemical modifications on the antibacterial activity could be separated from the effect on its efflux and permeation across the OM^3,32^. In agreement with previous studies^20,37^, we did not find a numeric correlation of the intracellular uptake of antibiotics and their antibacterial activities (Table 2). This lack of agreement between the two approaches is the result of technical differences: MIC values report on the concentration needed to kill growing bacterial cells, whereas LC/MS data report on time-dependent changes in the intracellular concentrations of compounds in non-growing cells. However, both datasets are usable to derive permeation rules when concentrations are measured in wild type, hyperporinated and efflux-deficient cells^24,30^. Future studies will be focused on identification of properties that are needed to develop models in which antibacterial activities of antibiotics could be predicted based on the permeation studies.

## Methods

### Materials

MS grade water, acetonitrile and formic acid were used for the LC–MS method. Radioactively labeled compounds [^3^H]-oxymetazoline hydrochloride (specific activity 1.5 Ci/mmol), [^14^C]-nepafenac (specific activity 50.0 mCi/mmol), [^14^C]-efavirenz (specific activity 56.7 mCi/mmol), [^3^H]-delavirdine (specific activity 1.2 Ci/mmol), [^3^H]-tetracycline (specific activity 17.9 Ci/mmol), [^14^C]-ciprofloxacin (specific activity 58.8 mCi/mmol), [^3^H]-metoprolol (specific activity 29.5 Ci/mmol) and their unlabeled analogs were purchased from Moravek Biochemicals Inc. (CA, USA). Other compounds were purchased from ChemDiv (CA, USA), MicroSource, Otava and TimTec or requested from National Cancer Institute. Compound registration and assay data management were carried out by Collaborative Drug Discovery.

### Strains and growth media

In all experiments, *P. aeruginosa* PAO1-Pore (PAO1 *att*Tn7::P_LAC_-*fhuA*) and PΔ3-Pore (Δ*mexAB* Δ*mexCD* Δ*mexXY att*Tn7::P_LAC_-*fhuA*) strains were used^29^. Strains were routinely grown in Luria–Bertani (LB) or MOPS-M9 medium at 37 °C with aeration. MICs shown in Tables 1 and 2 were determined by a two-fold dilution method in LB broth and MOPS-M9 medium, respectively, following the previously described protocol^30^.

### Intracellular accumulation of radioactively labeled compounds

PAO1-Pore and PΔ3-Pore were grown at 37 °C overnight with shaking at 225 rpm in LB medium. Cells were sub-cultured (50–100 mL) 1:100 in fresh LB broth in two flasks per strain. At OD_600_ 0.3 (~ 1.5 to 2 h of incubation), the expression of the pore was induced with 0.1 mM IPTG (final concentration) in one of the flasks and cells were grown for additional 1 to 1.5 h (OD_600_ 1- 1.5). Cells were collected by centrifugation at room temperature (RT) and washed in PMG Buffer (50 mM KPi, pH 7.0, 1 mM MgSO_4_, 0.4% glucose, prepared fresh) and concentrated 20x (i.e., 100 mL culture resuspended in 5 ml of PMG Buffer with final OD_600_ ~ 20–30). Assays were performed in 96-well 1.0 µm Glass Fiber Type B filters plates (Millipore). Cells were incubated with indicated concentrations of compounds, 100 µL cell aliquots were withdrawn after 0.5, 1, 2, 4, 6, 8, 16, and 32 min incubation at RT and collected by vacuum filtration onto filters.

Because compounds that penetrate the cells but do not have intracellular binding sites could be washed out by buffer, we first analyzed how washing of cells affects retention of radioactively labeled compounds (Fig. S1). We found that washing of filtered cells with buffer differentially affects compounds with variable intracellular affinities. Therefore, the washing step was avoided. Filter plates were dried, removed from the vacuum manifold and left in the hood overnight to dry completely. Filters were punched out from the plates, placed into 1 mL of scintillation counter solution and radioactivity counted using Tri-Carb 2810 TR Liquid Scintillation Analyzer (Perkin Elmer).

### Preparation of cells for LC–MS analysis

PAO1-Pore and PΔ3-Pore were grown at 37 °C overnight with shaking at 225 rpm in 50 mM MOPS-M9 (pH 7.2) Minimal Medium supplemented with 1% glycerol^42,43^. Importantly, a vancomycin spot assay showed that hyperporination of the OM was efficient in this medium and cells became highly susceptible to this antibiotic when incubated in the presence of inducer (Fig. S5).

The next day, cells were sub-cultured (1.5:100) in fresh MOPS-M9 medium and grown for 16 h. The cultures were then split into two flasks and one of them was induced with 0.1 mM IPTG. Cells incubated for additional 4 h until OD_600_ reached ~ 1.0 and harvested by centrifugation at 3,275xg for 20 min in RT, washed and concentrated 10X in the spent medium. Assays were performed in 96-well plates in a final volume of 200 mL. Cells were incubated with 10, 20, 40, 80 µM of compounds and after 1- and 40-min incubation at RT, 100 µL cell aliquots were collected by vacuum filtration onto 1.0 µm Glass Fiber Type B filters. For no-cell controls, compounds were diluted to 10, 20, 40, 80 µM final concentration in the same medium, incubated for 1 min and 40 min and filtered onto the same plates. The cell loaded and cell-free filters were dried, removed from plates and placed into 100% methanol at -80 °C for at least 10 min. Filter-bound material was extracted by water bath sonication for 1 min. Cell and filter debris were separated by ultracentrifugation at 45,000 rpm (TLA55 rotor, Beckman) for 8 min at 16 °C and the pellet re-extracted with 80% methanol in water by sonication for 15 min followed by ultracentrifugation at the same conditions. Supernatants from two extractions were combined. For the compound quantification, 5 µL of solution was analyzed in triplicate.

Calibration curves for MS analyses of compounds were generated in the same experiment by mixing compound at increasing concentrations with the sonicated PAO1 cell extracts. For most of the tested compounds, a linear calibration curve was obtained using the LC–MS approach when compounds were analyzed in a pure solvent at concentrations in the 10 nM to 1000 nM range (Fig. S6). However, in cells extracted from filters, the detection of compounds at the external compound concentration below 10 mM was unreliable. Levofloxacin at 100 nM final concentration was used as an external control and added to each sample after extraction^14^. Azithromycin was used as a positive control in all experiments. We found that azithromycin is readily detectable in the presence of levofloxacin and the kinetics of its accumulation is reflective of differences in permeability properties of the four strains of *P. aeruginosa* (Fig. S7)*.*

We found that for 80% of the compounds (66 out 83) the signal on filters with cells was higher than from empty filters. For the remaining compounds, the signal from empty filters was similar to or higher than from filters with cells and these compounds were excluded from further analyses.

### LC–MS quantification of intracellular accumulation of compounds

200 mL of samples supplemented with 100 nM levofloxacin as external standard were transferred into glass vials and 10 μL of samples and standards were injected into the UPLC in triplicates. A UPLC/HRMS system Agilent 1290 Infinity UPLC coupled to an Agilent 6545 quadrupole time-of-flight mass spectrometer was used to quantify the concentrations of compounds. Samples were processed in positive ion mode. An Eclipse plus C18 Rapid Resolution HD column (1.8 μm, 2.1 × 50 mm, Agilent, USA) was used for separation. The flow rate was 0.6 mL/min using the following gradient: 0 to 1 min (0- 20% ACN), 1 to 4 min (20- 80% ACN), 4 to 5.1 min (80–100% ACN), 5.1 to 6.30 (100% ACN). Solvents contained 0.1% formic acid to promote positive ion formation in the electrospray. The MS parameters were as follows: ion-source gas temperature, 325 °C; capillary voltage, 4000 V; fragmentor voltage, 180 V; *m/z* range, 50–1100; data acquisition rate, 4 GHz; and 2 spectra were recorded per second. Data were analyzed using Agilent Masshunter workstation quantitative analysis v. B.10 software.

### Determination of volumes of loaded cells and empty filters

To determine the cell-free volume of filters and the volume of filters occupied by loaded cells, we used microspheres (20 nm) labeled with Nile Red fluorophore (FluoroSpheres, ThermoFisher Scientific, Cat# F8784). These microspheres are too large to penetrate the cells and bind to cell surfaces and they are expected to be retained only by the cell-free volume of the filters. FluoroSpheres in the final concentrations of 10, 20, 40 and 80 mg/mL were incubated for 40 min without and with PAO1 cells (1.0 OD) in 100 mL of MOPS-M9 medium. FluoroSphere solutions with and without cells were vacuumed onto the filters, filter dried, removed from plates and fluorospheres were extracted twice with 0.3 mL HPLC-grade water. Fluorescence was quantified using a microplate reader and converted into amounts retained on filters. The retained volumes were determined from the slopes of the amounts of FluoroSpheres retained by filters with and without cells plotted as a function of concentration of solutions (Fig. S1). The difference in volumes is the volume displaced by cells.

### Synthesis of trisubstituted piperazin-2-one derivatives

**Figure 7:**
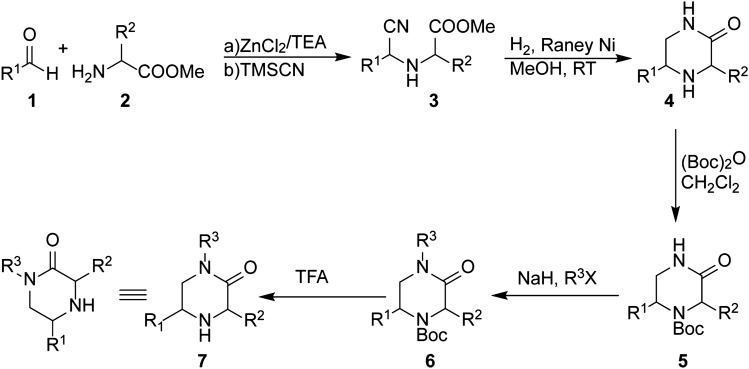
Synthesis of trisubstituted piperazin-2-one.

Our synthetic approach for building the trisubstituted piperazin-2-one derivatives involves two key steps: a modified Strecker reaction for the synthesis of cyanomethylamino pseudodipeptides (**3**), followed by a reductive cyclization to provide the core piperazin-2-ones (**4**), which could be functionalized accordingly to yield desired analogs. The synthesis commenced by treatment of the aldehyde **1** with the amino acid methyl ester **2** and TMSCN in presence of catalytic ZnCl_2_^44^. The pseudodipeptide **3** was obtained as an epimeric mixture at the stereogenic center of the peptide bond surrogate [(1*R*)/(1*S*) 1:3], which could not be resolved by chromotography. Reductive cyclization of pseudodipeptides **3** by catalytic hydrogenation resulted in in situ lactamization, in the presence of Raney Ni as the catalyst and led to the 5-substituted piperazin-2-ones **4** (Fig. 7) in 52–76% yield. The diastereomeric ratio remained unchanged in this reductive cyclization [(*R*)/(*S*) 1:3]. Boc protection of the secondary amine (**5**) was followed by alkylation of the secondary amide in the presence of NaH in DMF and the requisite alkyl halides to provide (**6**). Removal of the Boc-group in the presence of TFA yielded the final trisubstituted piperazin-2-ones **7**.

### Computational and machine learning methods

#### Cheminformatics

The chemical diversity of our compounds was compared to that of five other libraries and intracellular accumulation studies, including the Shared Platform for Antibiotic Research and Knowledge (SPARK) Public Projects Data^1^ (archived by Collaborative Drug Discovery Vault), FDA-approved drugs^36^, and small molecule permeability in bacteria studies by Richter et al.^16^, Davis et al.^15^, and Iyer et al^20^. In this comparison, nine features were used to describe each molecule: the molecular weight, logP, the number of hydrogen bond donors, the number of hydrogen bond acceptors, log D at pH 7.4, the topological polar surface area, Fsp3, the heavy atom count and the number of rotatable bonds. Molecular features for molecules from the four intracellular accumulation studies were calculated using the Marvin suite from ChemAxon^45^. All molecules were filtered to retain only those with molecular weights between 200 and 1,500 g/mol and to remove ions and peptides. Standardization and principal component analysis were then applied to the data points using scikit-learn version 0.24.1^46^. To illustrate how much each feature contributes to a particular principal component, PCA loadings are labeled in Fig. 2B. PCA loadings are defined as the coefficients of the linear combination of the original variables from which the principal components are constructed. Loading vectors were scaled by a factor of 7 for clarity. Figures were generated by matplotlib (version 3.3.4)^47^ and seaborn (version 0.11.1)^48^.

#### Physicochemical property calculation

Tautomers and protonation states at pH 7.4 for each compound were predicted using Marvin from ChemAxon^45^. SMILES strings were converted to 3D coordinates and a maximum of 50 low-energy conformers within an energy window of 10 kcal/mol were generated with the *RDKitGenerateConformers.py* script from MayaChemTools^49^ using the ETKDG conformer generator^50^ and the MMFF94s force field^51^. Both 2D and 3D descriptors were calculated with the *RDKitCalculateMolecularDescriptors.py* script from MayaChemTools and then averaged for use in subsequent machine learning analyses. RDKit version 2020.09.1 was used in all cases.

We also adapted a previously published protocol for computing force field parameters and running molecular dynamics (MD) simulations of antimicrobial compounds to extract physicochemical properties from the resulting trajectories^52^. Using the structures from the tautomer prediction as starting coordinates, we optimized the geometries of each compound at the quantum chemical density functional theory B3LYP/6-31G(d,p)^53–55^ with the polarizable continuum solvent model^56^ using Gaussian16 revision A.03^57^. Partial charges were generated using the restrained electrostatic potential (RESP)^58^ method in the *antechamber* utility^59^ and 100 ns MD simulations were then performed with AMBER 16^60^ using general AMBER force field (GAFF) parameters and the TIP3P water model^61^ with 0.1 M KCl. Additional details on the MD simulations have been described previously^52^. The PLUMED plugin^62^ was used to calculate several shape descriptors, including asphericity, acylindricity, k^2^, and the radius of gyration. These descriptor values were then averaged over the MD simulation and used for machine learning analysis. In total, 217 descriptors were calculated from MD simulations and with RDkit.

#### Selection of descriptors

The initial list of 217 descriptors was manually inspected to select only those that represent integral physicochemical or topological properties of a molecule or generic molecular fingerprints. The selected 47 descriptors were then clustered and analyzed by a modified elbow-method. Namely, the unexplained variance was calculated as a function of the number of clusters, fit to a three-segmented straight line, and the larger elbow used as a cutoff. Descriptors closest to the centers of the identified 30 clusters were subsequently used in ML analyses.

#### Random forest classification

A procedure consisting of data standardization, oversampling, and random forest (RF) classification was developed for this task. All procedures were performed with Python 3.8.8 in a Jupyter Notebook environment (Available at https://github.com/yshen25/EPI_ML) with scikit-learn version 0.24.1^63^ and imbalanced-learn version 0.8.0^64^. Of the 66 compounds, 25 were identified as permeators and the remaining 41 were labeled as non-permeators (Fig. 5). Because the dataset is imbalanced, the use of an oversampling technique was expected to be beneficial. Based on the mapping result (Fig. 6), the permeators belong to six different clusters in feature space, so common techniques such as SMOTE^65^ and ADASYN^66^ may produce noise points because synthetic data points may be situated in overlapping class regions. To overcome this issue, k-means SMOTE^67^ was applied. By giving more weight to sparse minority areas and avoiding overlapping zones, k-means SMOTE is expected to be better at overcoming skewness. Physicochemical properties were standardized to have mean and standard deviation of 0 and 1.

To obtain increased accuracy in the RF model, three hyperparameters were tuned using an exhaustive grid search. Hyperparameters included the number of trees in the random forest (*n_estimators*), the minimum number of samples required at a leaf node (*min_samples_leaf*), and the maximum number of features considered at each split (*max_features*). The hyperparameter combination with the highest accuracy was then selected for model training and testing. The performance of the final model was evaluated by leave-one-out cross validation. The 95% confidence interval was calculated by performing five-fold stratified cross validation repeated five times. Model performance was evaluated by the following scoring metrics. The *accuracy* describes the portion of samples that are correctly classified and is defined as follows:

$$accuracy = \frac{true\;positives + true\;negatives}{{all\;samples}}$$

*Precision* is the proportion of true positives among all predicted positives, also called *positive predictive value*:

$$precision = \frac{true\;positives}{{true\;positive + false\;positives}}$$

*Recall* is the proportion of true positives among all actual positives, also called *sensitivity*.

$$recall = \frac{true\;positives}{{true\;positives + false\;negatives}}$$

The *F1* score assesses the balance between *precision* and *recall:*

$${\text{F}}1 = 2 \times \left( {precision \times recall} \right)/\left( {precision + recall} \right)$$

The receiver operating characteristic (ROC) curve plots the true positive rate versus the false positive rate. The area under the ROC curve, AUROC, quantifies the ability of the model to discriminate between classes, with 1.0 corresponding to a perfect classifier and 0.5 corresponding to random.

The Gnomic program was developed using MATLAB 2019a with the Statistics and Machine Learning toolbox^68^.

## Supplementary Information

Supplementary Information 1.

Supplementary Information 2.

## Data Availability

All data generated or analyzed during this study are included in this published article and its supplementary information files.
