# Author Correction: Property space mapping of *Pseudomonas aeruginosa* permeability to small molecules

**DOI:** 10.1038/s41598-023-35960-5

**Published:** 2023-06-01

**Authors:** Inga V. Leus, Jon W. Weeks, Vincent Bonifay, Yue Shen, Liang Yang, Connor J. Cooper, Dinesh Nath, Adam S. Duerfeldt, Jeremy C. Smith, Jerry M. Parks, Valentin V. Rybenkov, Helen I. Zgurskaya

**Affiliations:** 1 Department of Chemistry and Biochemistry, University of Oklahoma, 101 Stephenson Parkway, Norman, OK 73019 USA; 2 Graduate School of Genome Science and Technology, University of Tennessee, Knoxville, TN 37996 USA; 3 Biosciences Division, Oak Ridge National Laboratory, Oak Ridge, TN 37831 USA; 4 Department of Medicinal Chemistry, University of Minnesota, 717 Delaware St. SE, Minneapolis, MN 55414 USA; 5 Department of Biochemistry and Cellular and Molecular Biology, University of Tennessee, Knoxville, TN 37996 USA

Correction to: *Scientific Reports* https://doi.org/10.1038/s41598-022-12376-1, published online 17 May 2022

The original version of this Article contained an error in the spelling of the author Dinesh Nath which was incorrectly given as Dinesh Nash.

The original Article and accompanying Supplementary Information 1 file have been corrected.

