# Supplementary material for: Property space mapping of Pseudomonas aeruginosa permeability to small molecules
Source: Sci Rep. 2022 May 17;12:8220. doi: 10.1038/s41598-022-12376-1 (PMC9114115; doi:10.1038/s41598-022-12376-1)
Supplement: Supplementary file 1 — Supplementary Information 1. [file 41598_2022_12376_MOESM1_ESM.pdf]

Property space mapping of *Pseudomonas aeruginosa* permeability to small molecules

Inga V. Leus[1], Jon W. Weeks[1], Vincent Bonifay[1], Yue Shen[2], Liang Yang[1], Connor J. Cooper[3], Dinesh Nath[1], Adam S. Duerfeldt[4], Jeremy C. Smith[2,5], Jerry M. Parks[3], Valentin Rybenkov[1,*] Helen I. Zgurskaya[1,*]

Table of contents:

Table S1. Structures, intracellular accumulation levels and clusters of analyzed library of 66 compounds. (see Table_S1.xlsx).

Table S2. MICs of antibiotics in four strains of *P. aeruginosa* grown in LB broth.

Table S3. Coordinates of the principal components for Figure 3A.

Table S4. Top descriptors and their definitions.

Figure S1. The effect of cells on retention of compounds.

Figure S2. Kinetics of Intracellular accumulation of [^{14}C]-labeled ciprofloxacin.

Figure S3. Kinetics of Intracellular accumulation of [^{3}H]-labeled oximetazoline.

Figure S4. Violin plots of compounds from two chemical libraries and four studies.

Figure S5. Vancomycin susceptibility spot assay with *P. aeruginosa* hyperporinated strains grown on the minimal medium.

Figure S6. LC-MS representative calibration curves.

Figure S7. Concentration and time- dependent accumulation of azithromycin in four strains of *E. coli*.

SI 1. Synthesis of trisubstituted piperazin-2-one derivatives.

Table S2. MICs of antibiotics in four strains of *P. aeruginosa* grown in LB broth[a].

**Antibiotic**	**PAO1**	**PAO1-Pore**	**P∆3**	**P∆3-Pore**
tetracycline	12.5	0.39-1.56	0.78	0.78
ciprofloxacin	0.19	0.09	0.05	0.02
doxorubicin	200	100	50	25
levofloxacin	1	0.25	0.0156	0.0039
norfloxacin	1	1	0.0625	0.0156
prulifloxacin	0.25	0.0625	0.0156	0.0039
nadifloxacin	6.25	0.39	0.097	0.0015
pazufloxacin mesylate	1	0.25	0.0156	0.0156
moxifloxacin hydrochloride	6.25	0.39	0.097	0.024
difloxacin hydrochloride	1.56	0.39	0.39	0.097
gatifloxacin	1	0.25	0.0625	0.0039
oxolinic acid	25	6.25	0.39	0.097
enrofloxacin	1	0.25	0.0625	0.0039
pefloxacine mesylate	1	0.25	0.0156	0.0039
sparfloxacin	1	0.25	0.0156	0.0039
lomefloxacin	1.56	1.56	0.097	0.024
linezolid	400	25	25	12.5

[a], sulfonamides do not have inhibitory activities in LB broth.

Table S3. Coordinates of the principal components for Figure 4A.

Uptake or uptake ratio	PC1	PC2	PC3	PC4	PC5	PC6	PC7	PC8
**PAO1, 1 min**	-0.13	0.68	-0.08	-0.08	0.00	0.16	0.07	0.69
**PΔ3-Pore/WT, 1 min**	0.41	-0.08	-0.36	0.19	0.63	-0.44	-0.08	0.25
**PΔ3-Pore /Pore, 1 min**	0.37	0.17	0.59	-0.16	0.34	0.30	-0.49	-0.08
**PΔ3-Pore / PΔ3, 1 min**	0.38	0.13	-0.26	0.70	-0.23	0.46	-0.06	-0.10
**PAO1, 40 min**	-0.13	0.68	-0.12	0.04	0.15	-0.27	0.02	-0.64
**PΔ3-Pore /WT, 40 min**	0.39	0.00	-0.42	-0.61	0.08	0.40	0.32	-0.18
**PΔ3-Pore /Pore, 40 min**	0.42	0.10	0.49	0.12	-0.10	-0.23	0.70	0.04
**PΔ3-Pore / PΔ3, 40 min**	0.43	0.12	-0.11	-0.24	-0.63	-0.43	-0.40	0.06
**Variance**	49.1	24.0	9.8	6.7	4.8	3.3	1.6	0.6

Table S4. Top descriptors and their definitions.

name	type	definition	class
NumRotatableBonds	1D	Number of Rotatable Bonds	Constitutional descriptor
NumAromaticRings	1D	Number of Aromatic rings	Constitutional descriptor
PMI2	2D	Second (largest) principal moment of inertia	Topological descriptor
FpDensityMorgan2	2D	Morgan fingerprint density	Topological descriptor
TPSA	2D	Topological polar surface area	Molecular property descriptor
avg_asphericity	3D	average asphericity over 100 ns MD trajectory	MD
avg_smallest_principal_rg	3D	average smallest principal radius of gyration over 100 ns MD trajectory	MD

RDKit descriptor definitions adapted from

https://cb.imsc.res.in/deduct/descriptors/eJaFhpBqbWtuZWt9

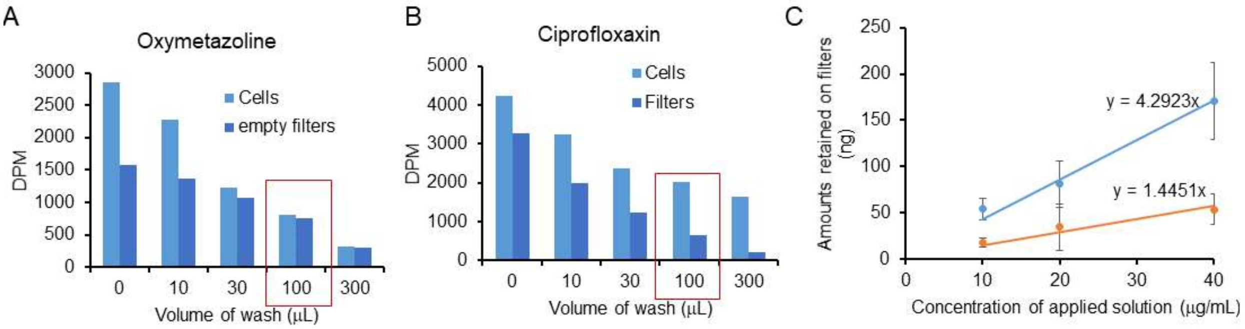

Figure S1. The effect of cells on retention of compounds. The permeant PΔ3-Pore cells were incubated with [^{14}C]-ciprofloxacin (**A**) and [^{3}H]-oxymetazoline (**B**) for 30 min, filtered onto 96-well filter plates and washed with increasing volumes of the incubation buffer. Empty filters and filters loaded with 100 μL containing 1.0 OD_{600} of cells retained different amounts of radioactivity, suggesting that cells accumulate both compounds. For [^{3}H]-oxymetazoline, 95% of the accumulated radioactivity was washed out of cells with the 0.1 mL wash. In contrast, [^{14}C]-ciprofloxacin with its high affinity DNA binding remained inside the cells even after the wash with three volumes. **C.** PAO1 cells were incubated with FluoroSpheres (20 nm) labeled with Nile Red and filtered onto 96-well filter plates along with solutions without cells. Filter loaded with cells (orange) and filters without cells (blue) were dried, removed from plates and fluorospheres were extracted as described in Methods. Fluorescence was quantified and converted into amounts retained on filters and plotted as a function of applied concentrations. Slopes of linear fits are volumes retained by filters with (1.5 μL) and without (4.3 μL) cells. The difference in volumes is the volume displaced by cells.

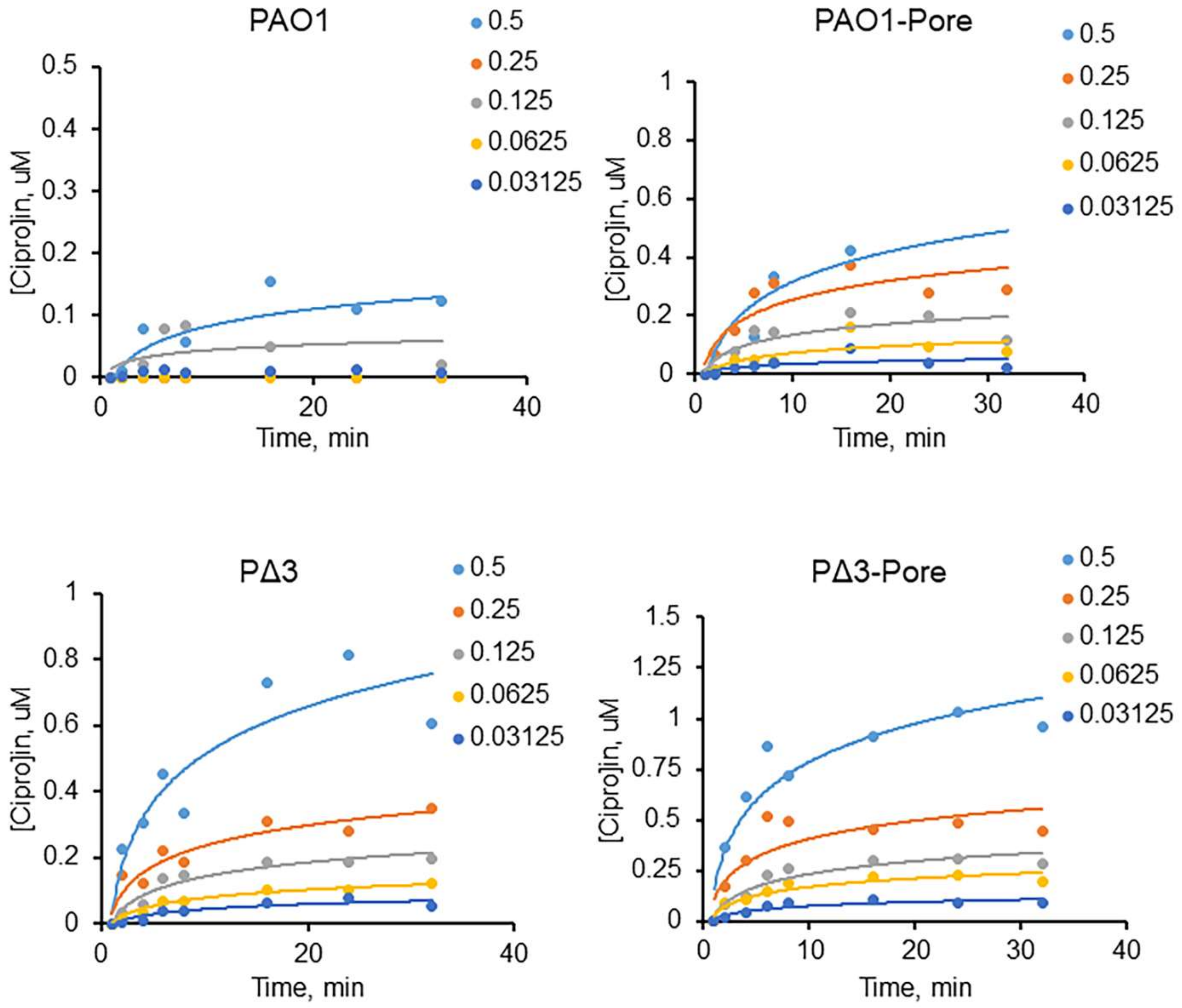

Figure S2. Kinetics of Intracellular accumulation of [^{14}C]-labeled ciprofloxacin. A representative experiment is shown. External concentrations of compounds (μM) are indicated in graph legends.

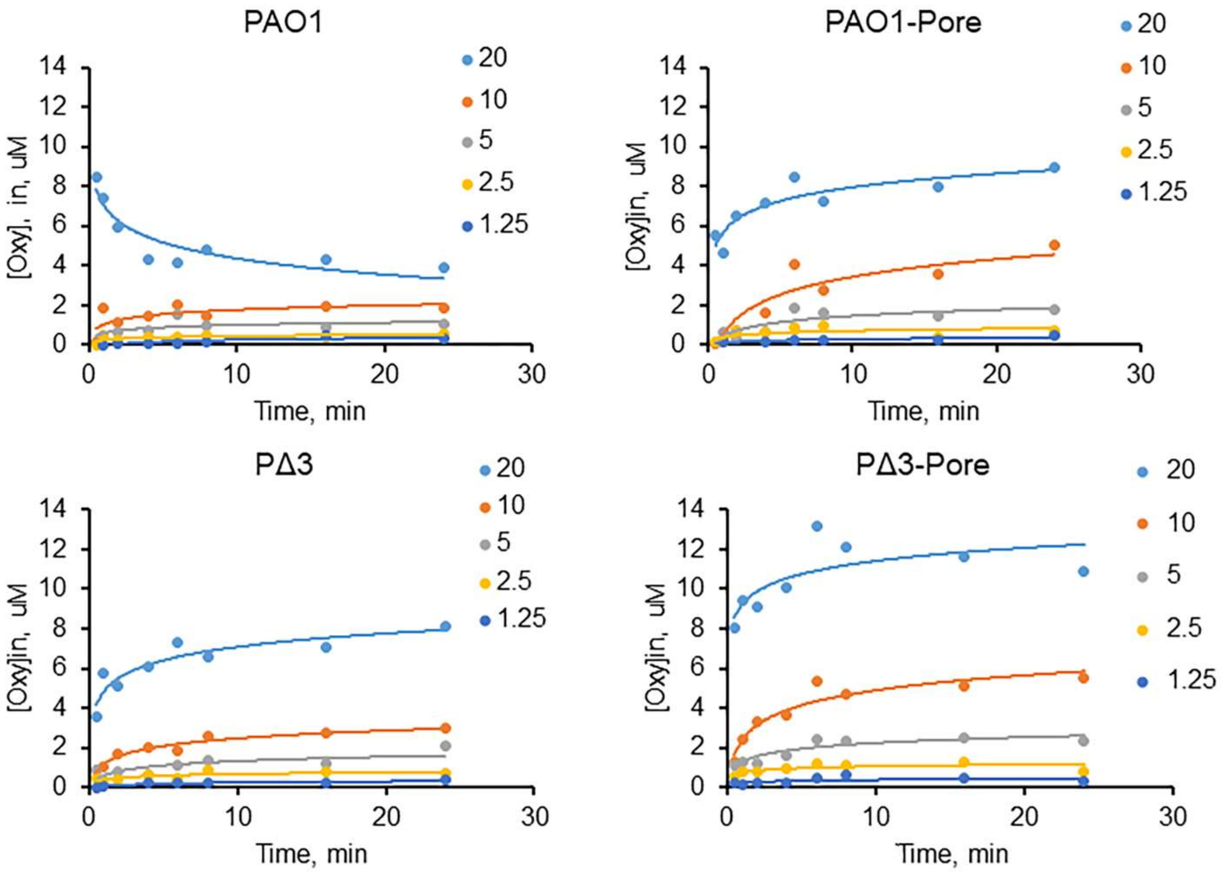

Figure S3. Kinetics of Intracellular accumulation of [^{3}H]-labeled oximetazoline. A representative experiment is shown. External concentrations of compounds (μM) are indicated in graph legends.

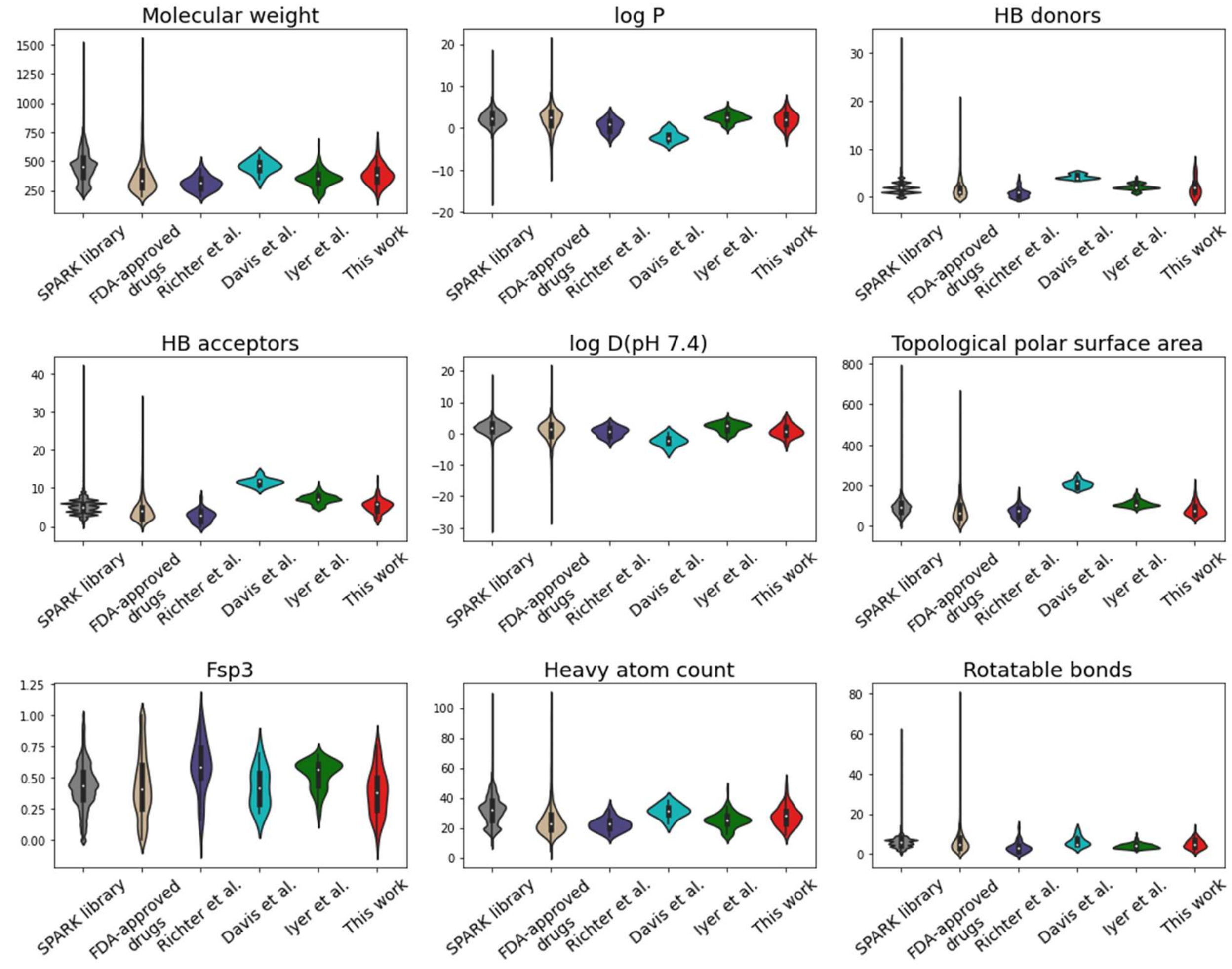

Figure S4. Violin plots of compounds from two chemical libraries and four studies showing the distribution of molecules among each of nine molecular features used to describe chemical space diversity.

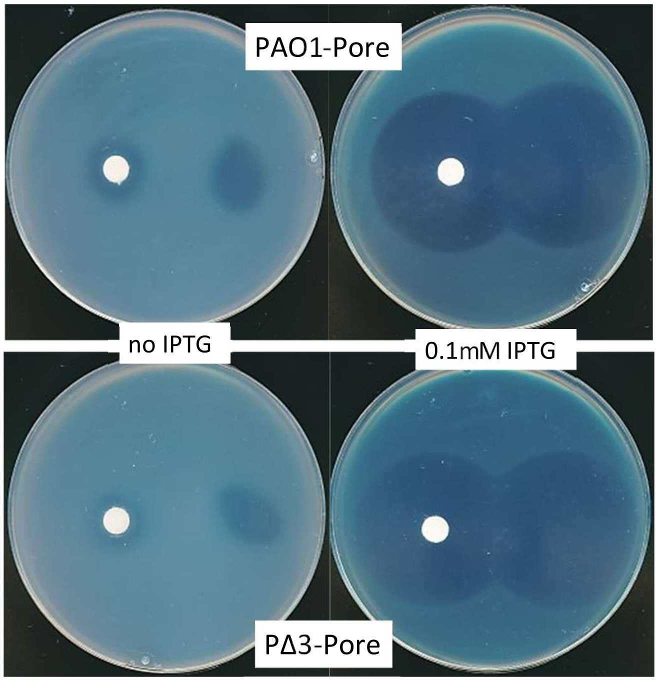

Figure S5. Vancomycin susceptibility spot assay with *P. aeruginosa* hyperporinated strains grown on the minimal medium.

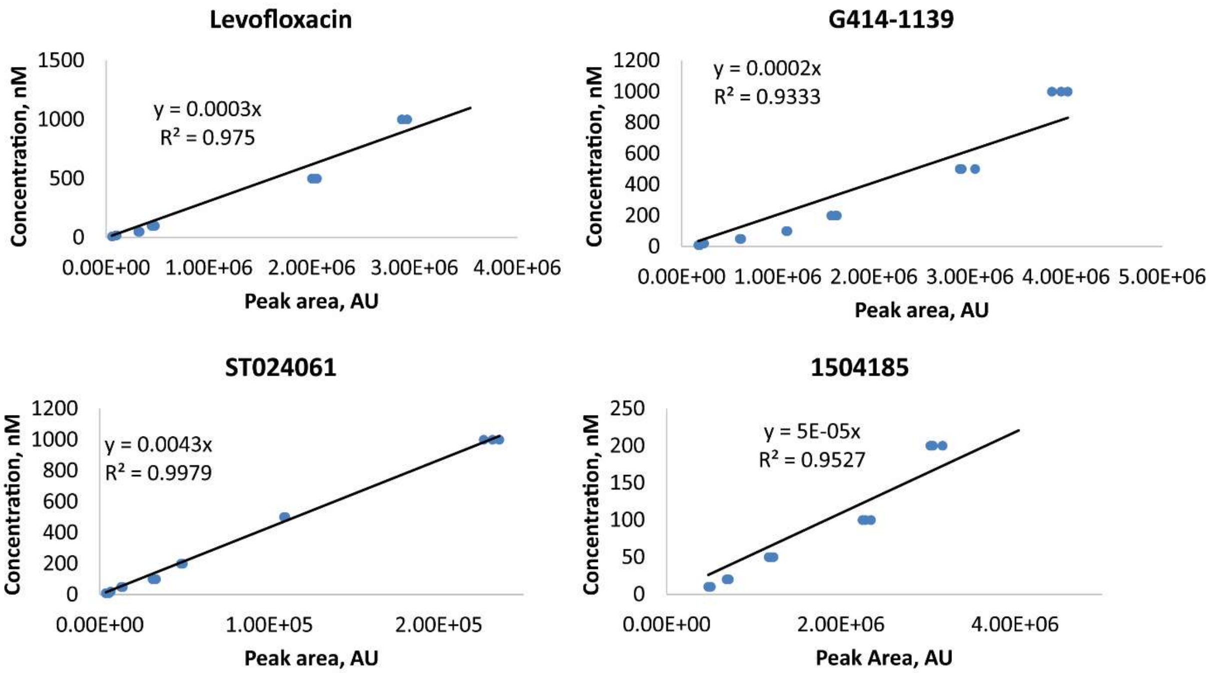

Figure S6. LC-MS representative calibration curves.

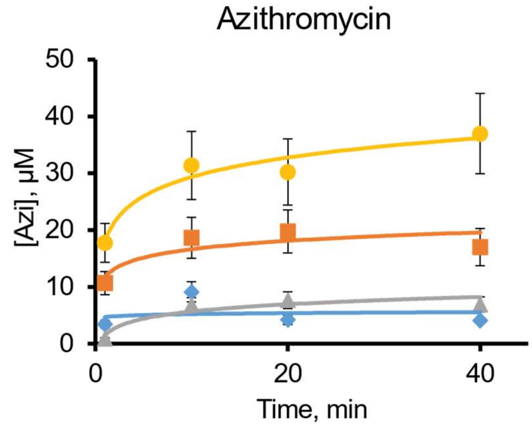

Figure S7. Concentration and time- dependent accumulation of azithromycin in four strains of *P. aeruginosa*: PAO1 (blue), PΔ3 (grey), PAO1-Pore (orange), PΔ3-Pore (yellow)

SI 1. Synthesis of trisubstituted piperazin-2-one derivatives.

(3S,5R)-5-(2-cyclohexylethyl)-3-(3,5-difluorobenzyl)piperazin-2-one (OU-0032508)

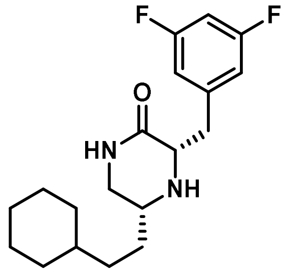

OU-0032508

^{1}H NMR (500 MHz, Chloroform-*d*) δ 6.82 (d, *J* = 7.3 Hz, 2H), 6.69 (t, *J* = 9.1 Hz, 1H), 6.20 (d, *J* = 4.3 Hz, 1H), 3.64 (dd, *J* = 9.4, 3.3 Hz, 1H), 3.40 (dd, *J* = 14.0, 3.4 Hz, 1H), 3.26 – 3.20 (m, 1H), 2.98 (t, *J* = 10.9 Hz, 1H), 2.85 (dd, *J* = 14.0, 9.0 Hz, 2H), 1.70 – 1.59 (m, 6H), 1.38 (q, *J* = 7.4 Hz, 2H), 1.14 (dd, *J* = 15.6, 8.6 Hz, 5H), 0.83 (d, *J* = 11.5 Hz, 2H). ^{13}C NMR (101 MHz, Chloroform-*d*) δ 171.8 171.1, 164.5, 164.4, 162.0, 161.9, 142.6, 142.5, 142.4, 112.5, 112.4, 112.4, 112.3, 112.2, 102.6, 102.3, 102.1, 59.9, 58.0, 53.1, 48.4, 48.3, 47.6, 38.1, 37.9, 37.8, 37.7, 33.9, 33.4, 34.0, 33.3, 31.1, 30.2, 26.7, 26.7, 26.4.

(3S,5S)-5-(2-cyclohexylethyl)-3-(3,5-difluorobenzyl)piperazin-2-one (OU-0032508)

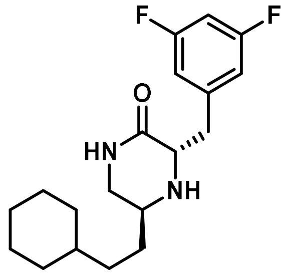

OU-0032508

^{1}H NMR (500 MHz, Chloroform-*d*) δ 6.94 (s, 1H), 6.78 (d, *J* = 7.1 Hz, 4H), 6.69 (td, *J* = 9.0, 4.5 Hz, 2H), 3.66 (dd, *J* = 10.4, 3.5 Hz, 2H), 3.32 – 3.26 (m, 2H), 3.18 (dd, *J* = 13.8, 3.6 Hz, 2H), 3.06 – 2.99 (m, 2H), 2.99 – 2.91 (m, 4H), 1.66 (t, *J* = 9.9 Hz, 10H), 1.37 (ddd, *J* = 23.8, 9.5, 5.0 Hz, 4H), 1.23 – 1.18 (m, 4H), 1.17 (s, 1H), 1.17 – 1.08 (m, 7H), 0.85 (dq, *J* = 10.7, 5.4, 4.9 Hz, 4H). ^{13}C NMR (101 MHz, Chloroform-*d*) δ 171.8, 164.5, 164.4, 162.0, 161.9, 142.7, 142.6, 142.5, 112.5, 112.5, 112.3, 112.3, 102.6, 102.3, 102.1, 58.0, 48.4, 47.6, 37.9, 37.9, 37.9, 37.7, 33.9, 33.4, 33.3, 30.2, 26.7, 26.4.

(3S,5S)-5-butyl-3-(3,5-difluorobenzyl)piperazin-2-one (OU-0032509)

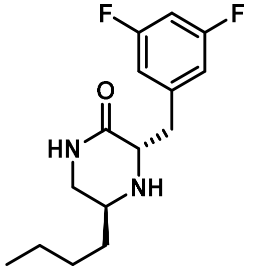

OU-0032509

^{1}H NMR (500 MHz, Chloroform-*d*) δ 6.82 (d, *J* = 7.3 Hz, 2H), 6.68 (t, *J* = 9.0 Hz, 1H), 6.55 (d, *J* = 4.3 Hz, 1H), 3.64 (dd, *J* = 9.3, 3.4 Hz, 1H), 3.39 (dd, *J* = 14.0, 3.3 Hz, 1H), 3.23 (d, *J* = 11.3 Hz, 1H), 2.97 (d, *J* = 11.0 Hz, 1H), 2.86 (td, *J* = 13.5, 7.8 Hz, 2H), 1.37 (t, *J* = 7.2 Hz, 3H), 1.31 – 1.19 (m, 4H), 0.86 (t, *J* = 7.1 Hz, 3H). ^{13}C NMR (101 MHz, Chloroform-*d*) δ 171.2, 164.5, 164.3, 162.0, 161.9, 142.6, 142.5, 142.4, 112.4, 112.4, 112.2, 112.2, 102.55, 102.3, 102.1, 59.9, 52.8, 48.2, 38.1, 38.1, 38.0, 33.4, 28.0, 22.8, 14.0.

tert-butyl (2S,6R)-6-(2-cyclohexylethyl)-2-(3,5-difluorobenzyl)-3-oxopiperazine-1-carboxylate (OU-0032510)

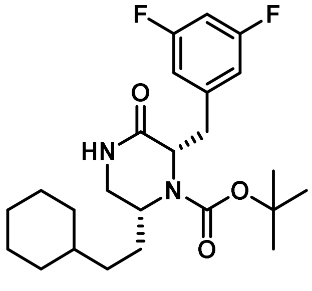

OU-0032510

^{1}H NMR (500 MHz, Chloroform-*d*) δ 6.83 – 6.77 (m, 2H), 6.73 – 6.65 (m, 1H), 3.74 – 3.63 (m, 2H), 3.39 (dd, *J* = 13.9, 3.5 Hz, 1H), 3.12 (t, *J* = 11.4 Hz, 1H), 2.92 – 2.79 (m, 2H), 1.70 – 1.59 (m, 5H), 1.40 (t, *J* = 8.0 Hz, 2H), 1.21 – 1.10 (m, 5H), 0.83 (d, *J* = 10.9 Hz, 2H). ^{13}C NMR (101 MHz, Chloroform-*d*) δ 170.2, 164.5, 164.3, 162.0, 161.9, 152.5, 142.1, 142.1, 142.0, 112.5, 112.4, 112.3, 112.3, 102.7, 102.5, 102.2, 83.6, 62.1, 53.0, 52.1, 38.5, 37.8, 33.4, 33.3, 31.3, 28.1, 26.7, 26.4.

tert-butyl (2S,6R)-4-allyl-2-benzyl-6-butyl-3-oxopiperazine-1-carboxylate (OU-0032511)

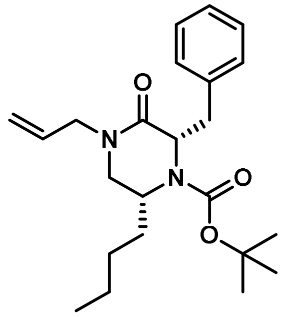

OU-0032511

^{1}H NMR (400 MHz, Chloroform-*d*) δ 7.30 – 7.24 (m, 2H), 7.21 (t, *J* = 10.3 Hz, 3H), 5.84 (ddt, *J* = 16.6, 11.1, 5.9 Hz, 1H), 5.29 (d, *J* = 2.5 Hz, 1H), 5.25 – 5.19 (m, 1H), 4.57 (d, *J* = 5.8 Hz, 2H), 4.52 (s, 1H), 3.50 (dd, *J* = 8.5, 5.9 Hz, 1H), 2.94 (dt, *J* = 13.3, 6.7 Hz, 3H), 2.77 (dd, *J* = 13.4, 8.6 Hz, 1H), 2.47 (d, *J* = 7.6 Hz, 1H), 1.56 (s, 1H), 1.25 (s, 9H), 0.86 (t, *J* = 6.6 Hz, 3H). ^{13}C NMR (101 MHz, Chloroform-*d*) δ 174.9, 156.2, 137.7, 131.8, 129.2, 128.4, 126.7, 118.8, 78.7, 65.5, 60.6, 55.8, 42.0, 40.4, 32.5, 29.7, 28.4, 28.0, 22.7, 13.9.

(3S,8aS)-3-((1H-indol-3-yl)methyl)hexahydropyrrolo[1,2-a]pyrazine-1,4-dione (OU-0032512)

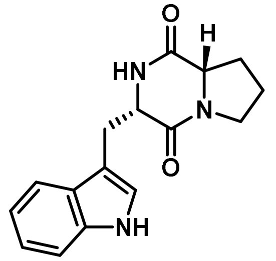

OU-0032512

^{1}H NMR (400 MHz, Methanol-d_4) δ 7.65 (d, *J* = 7.9 Hz, 1H), 7.37 (d, *J* = 7.9 Hz, 1H), 7.17 (t, *J* = 7.6 Hz, 1H), 7.10 (s, 2H), 6.68 (t, *J* = 9.4 Hz, 1H), 5.99 (d, *J* = 7.8 Hz, 2H), 4.26 (s, 1H), 3.85 – 3.77 (m, 1H), 3.26 (d, *J* = 3.9 Hz, 1H), 3.14 – 3.05 (m, 1H), 2.42 (d, *J* = 13.5 Hz, 1H), 0.99 (t, *J* = 11.5 Hz, 1H). ^{1}H NMR (500 MHz, Methanol-d_4) δ 168.0, 163.0, 132.5, 131.3, 126.1, 123.0, 120.6, 120.2, 113.5, 112.5, 112.5, 111.6, 71.0, 57.3, 41.6, 30.9, 27.9, 25.1.

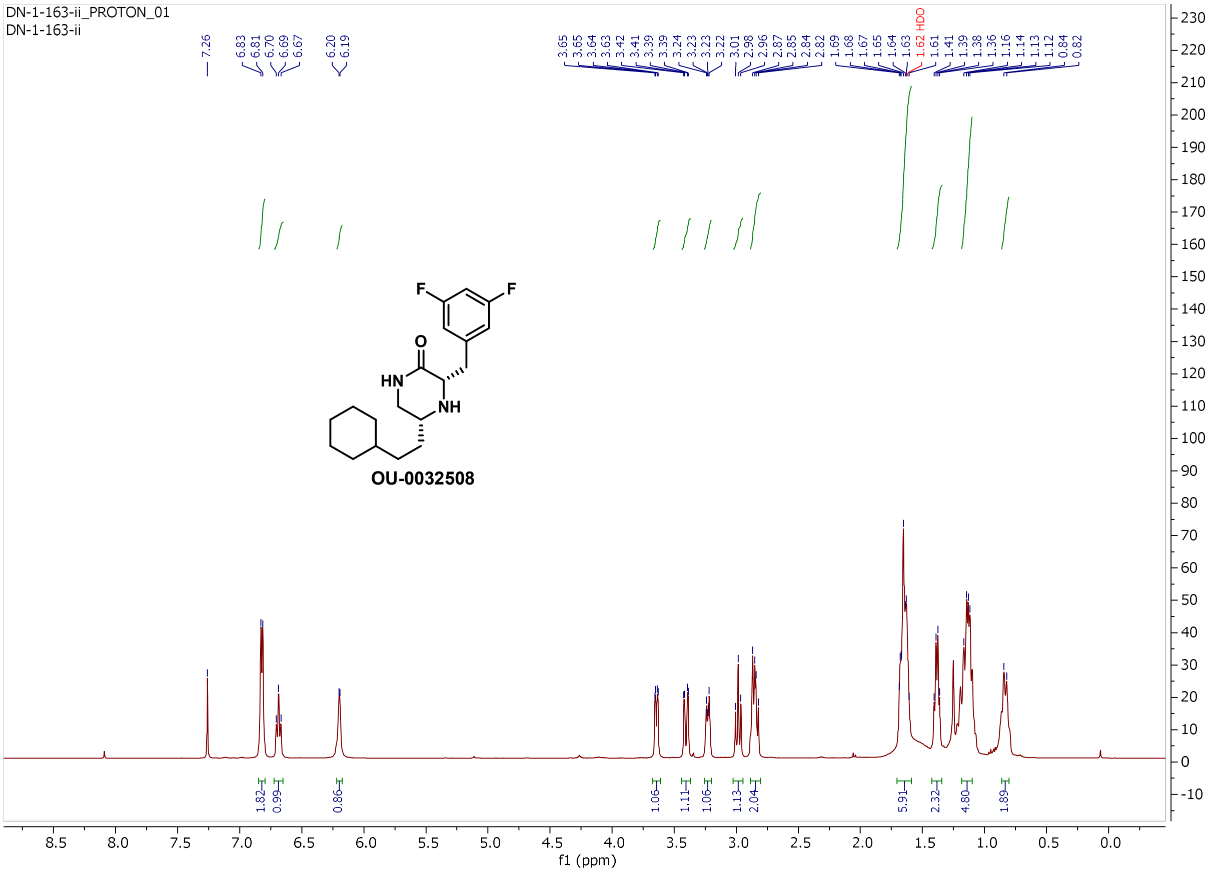
DN-1-163-ii_PROTON_01
DN-1-163-ii
7.26
6.83
6.81
6.70
6.69
6.67
6.20
6.19
3.65
3.65
3.64
3.63
3.42
3.41
3.39
3.39
3.24
3.23
3.23
3.22
3.01
2.98
2.96
2.87
2.85
2.84
2.82
1.69
1.68
1.67
1.65
1.64
1.63
1.62 HDO
1.61
1.41
1.39
1.38
1.36
1.16
1.14
1.13
1.12
0.84
0.82
F
F
O
HN
NH
OU-0032508
1.82
0.99
0.86
1.06
1.11
1.06
1.13
2.04
5.91
2.32
4.80
1.89
f1 (ppm)

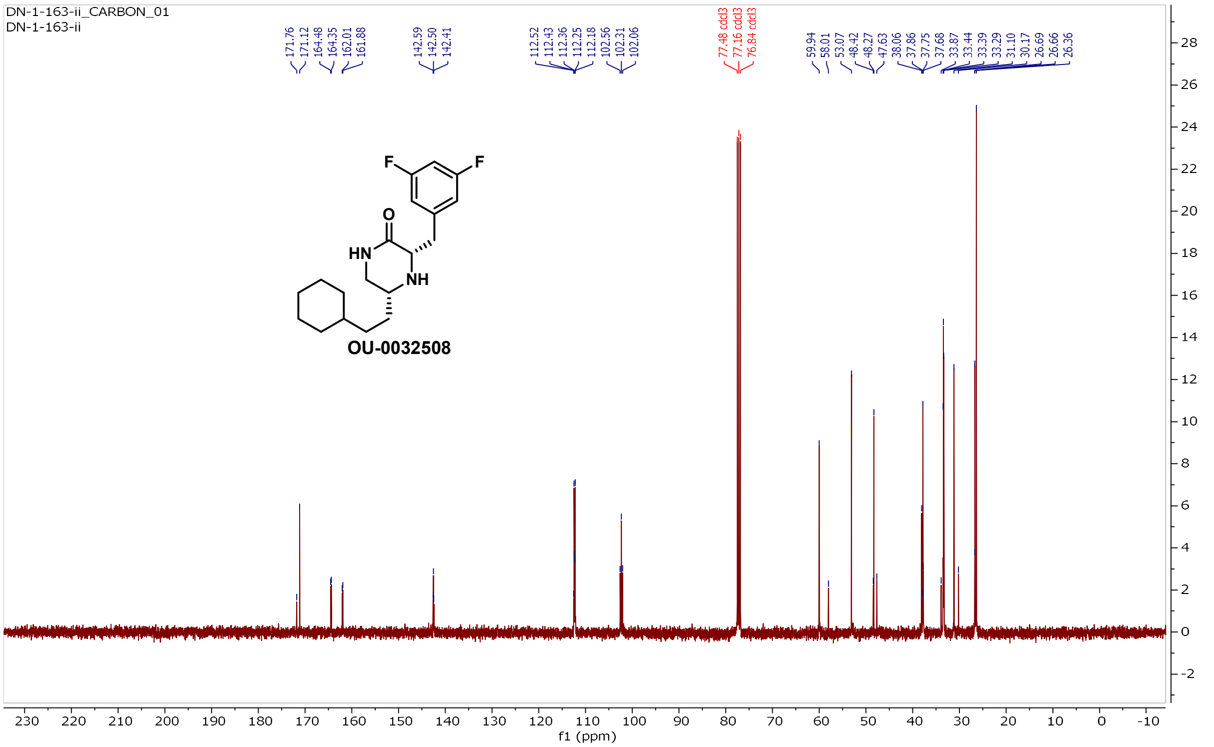
DN-1-163-ii_CARBON_01
DN-1-163-ii
171.76
171.12
164.48
164.35
162.01
161.88
142.59
142.50
142.41
112.52
112.43
112.36
112.25
112.18
102.56
102.31
102.06
77.48 cdcl3
77.16 cdcl3
76.84 cdcl3
59.94
58.01
53.07
48.42
48.27
47.63
38.06
37.86
37.75
37.68
33.87
33.44
33.39
33.29
31.10
30.17
26.69
26.66
26.36
F
F
O
HN
NH
OU-0032508
f1 (ppm)

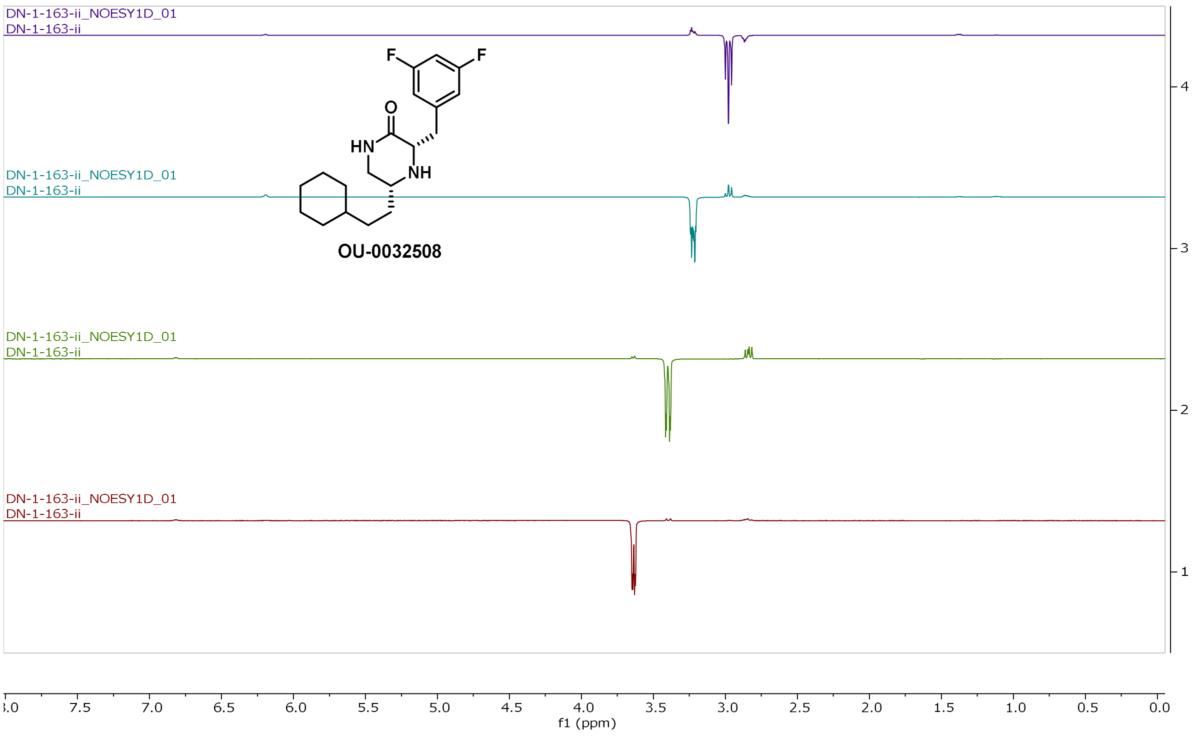
DN-1-163-ii_NOESY1D_01
DN-1-163-ii
OU-0032508
f1 (ppm)

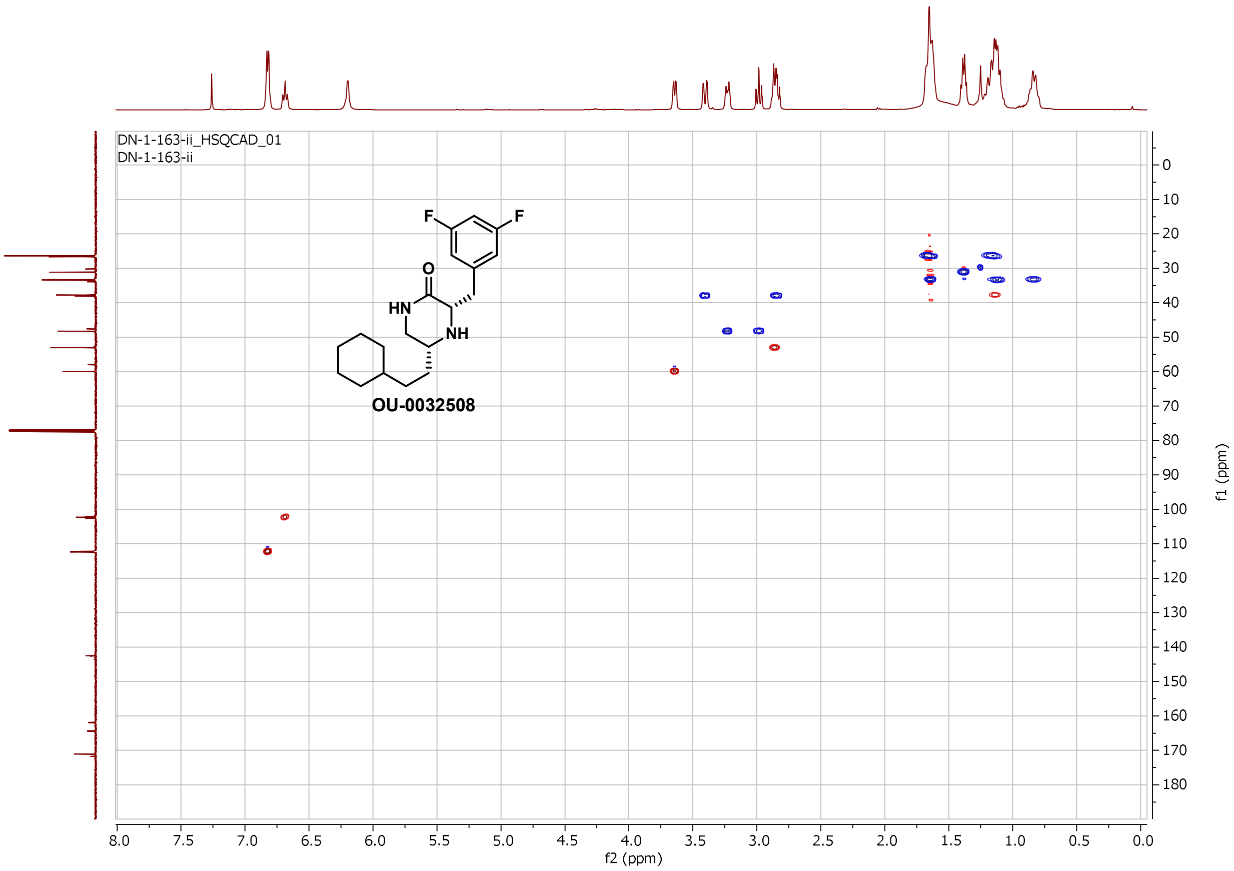
DN-1-163-ii_HSQCAD_01
DN-1-163-ii
OU-0032508
f2 (ppm)
f1 (ppm)

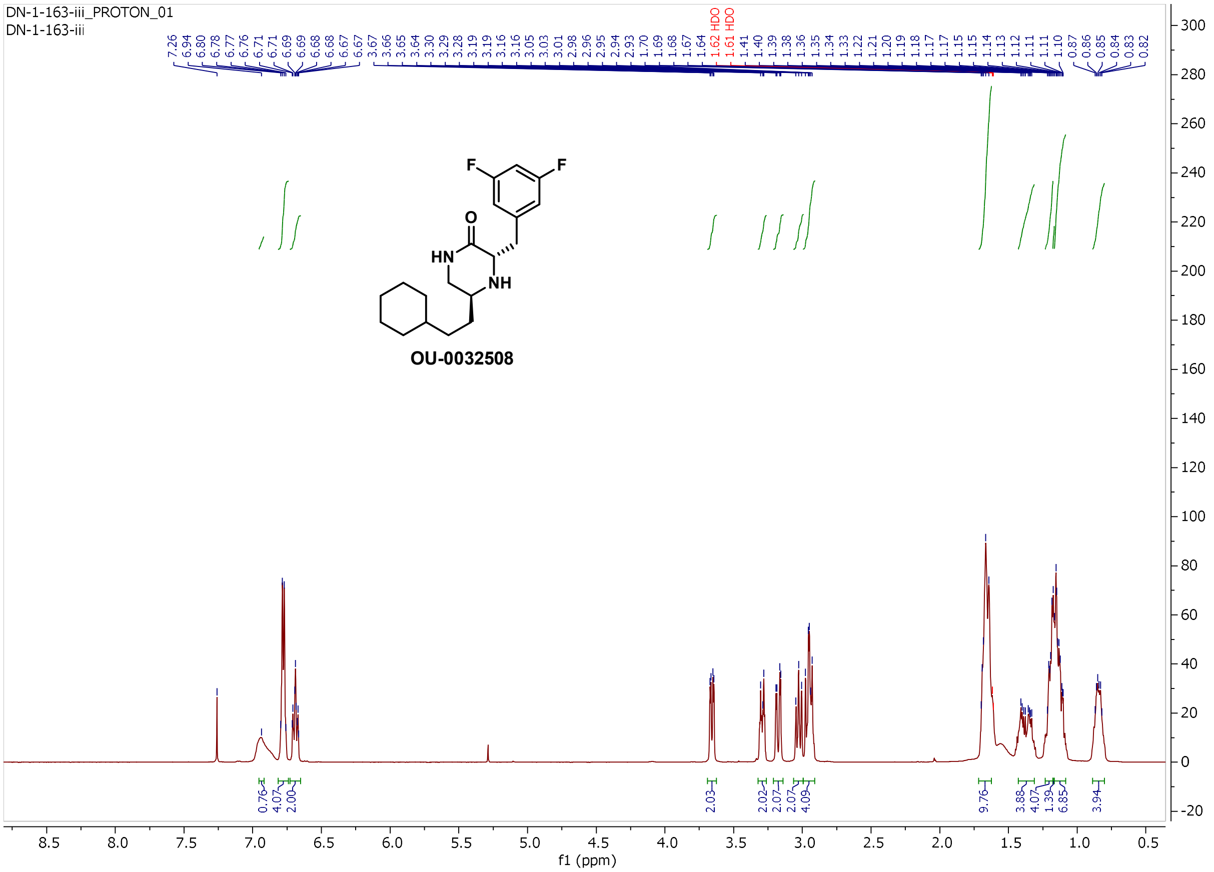
DN-1-163-iii_PROTON_01
DN-1-163-iii
OU-0032508
f1 (ppm)

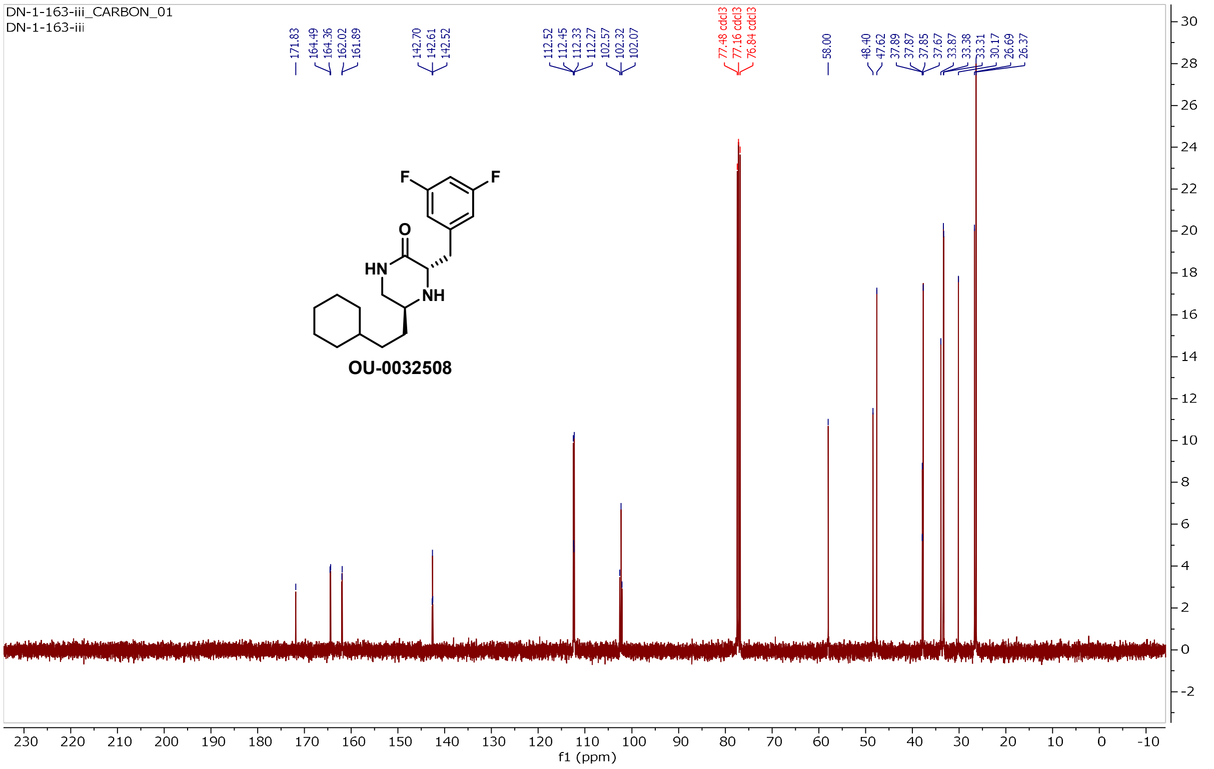
DN-1-163-iii_CARBON_01
DN-1-163-iii
OU-0032508
f1 (ppm)

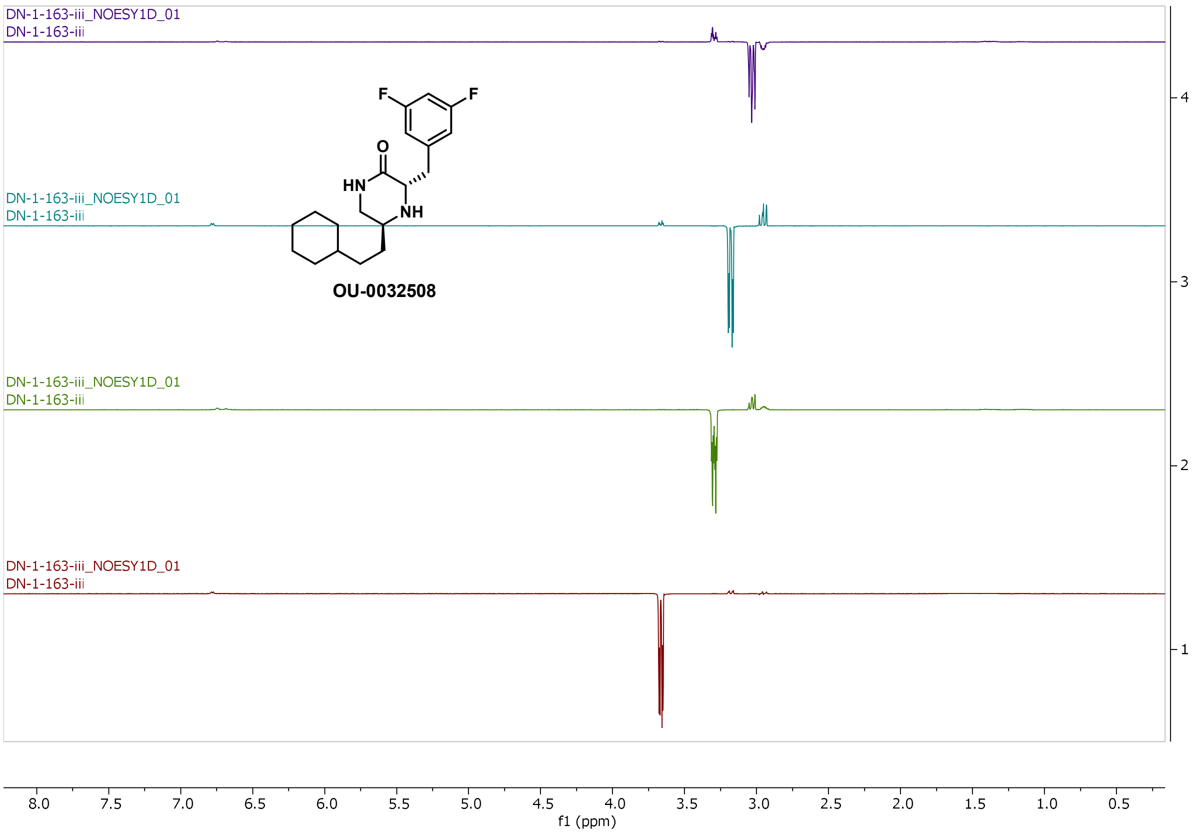

DN-1-163-iii_NOESY1D_01
DN-1-163-iii
OU-0032508
f1 (ppm)

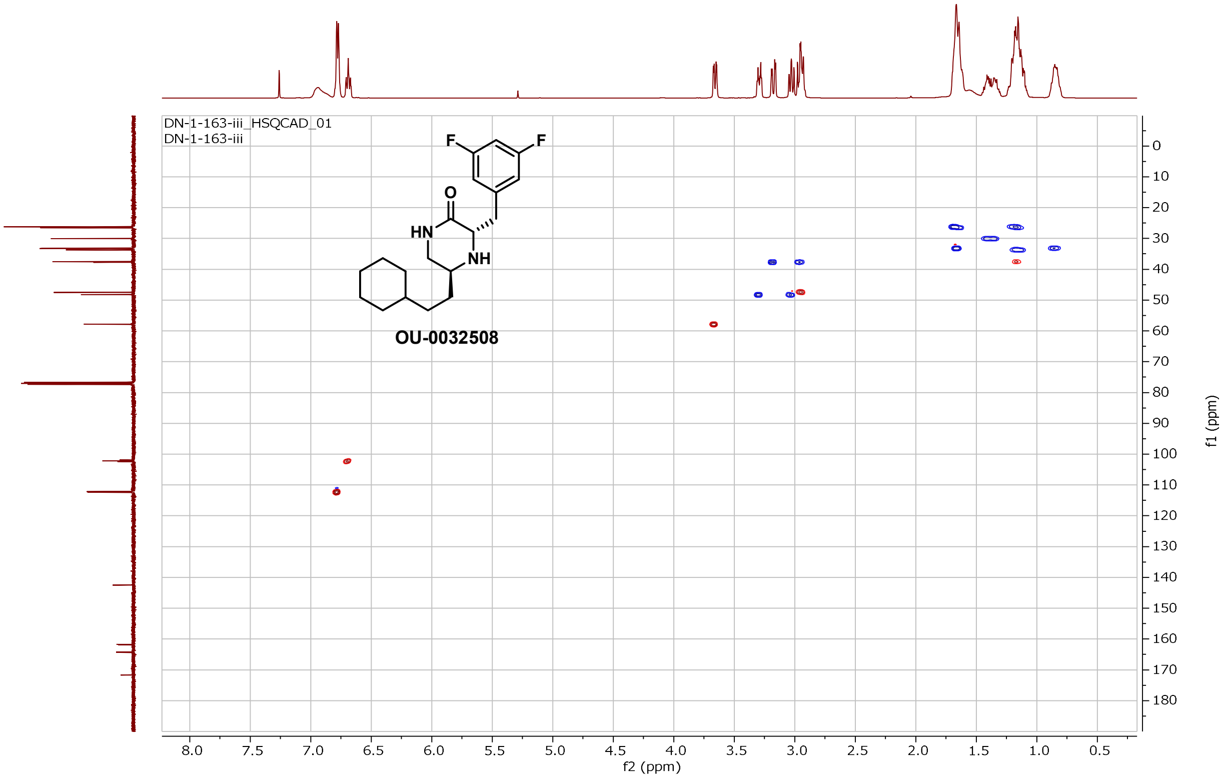

DN-1-163-iii_HSQCAD_01
DN-1-163-iii
OU-0032508
f2 (ppm)
f1 (ppm)

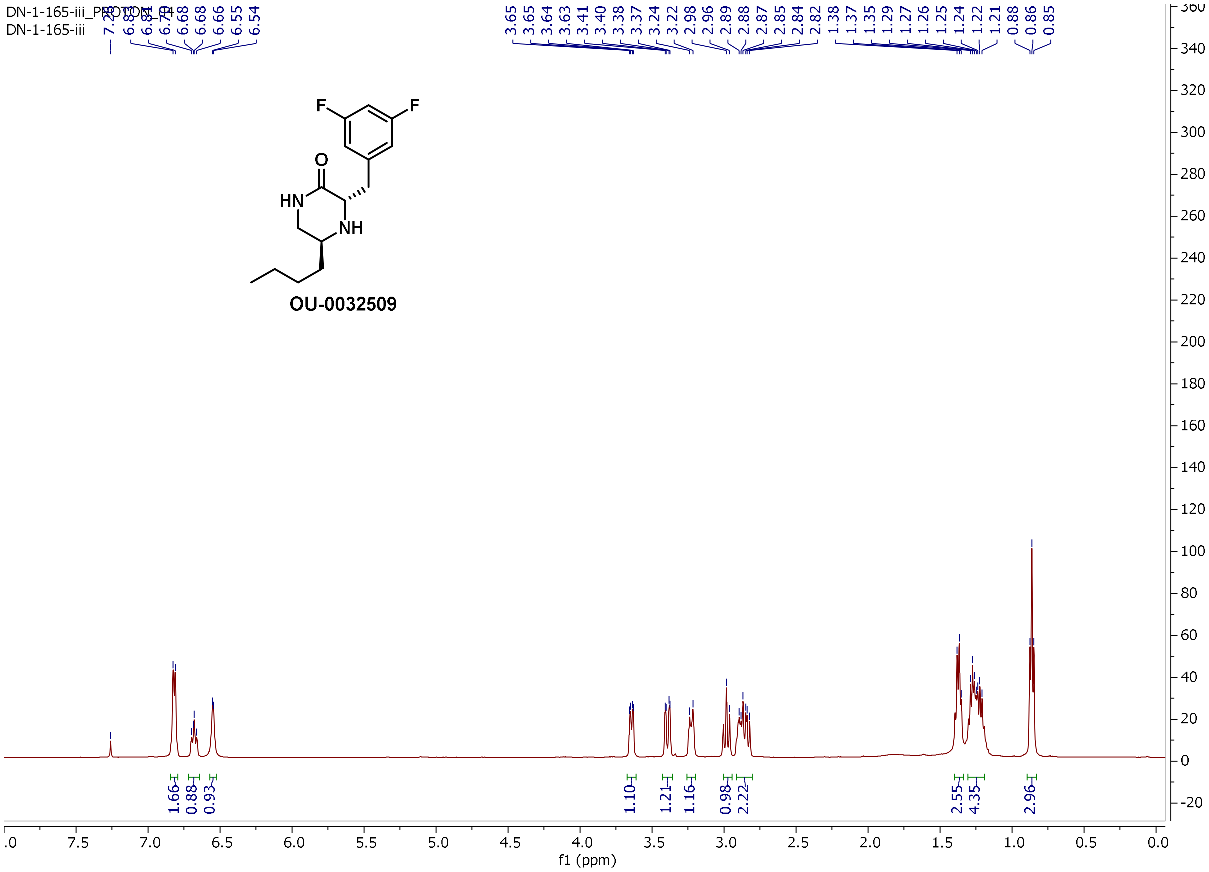
DN-1-165-iii_PROTON_01
DN-1-165-iii
3.65 3.65 3.64 3.63 3.41 3.40 3.38 3.37 3.24 3.22 2.98 2.96 2.89 2.88 2.87 2.85 2.84 2.82 1.38 1.37 1.35 1.29 1.27 1.26 1.25 1.24 1.22 1.21 0.88 0.86 0.85
6.68 6.68 6.66 6.55 6.54
1.66 0.88 0.93 1.10 1.21 1.16 0.98 2.22 2.55 4.35 2.96
OU-0032509
f1 (ppm)

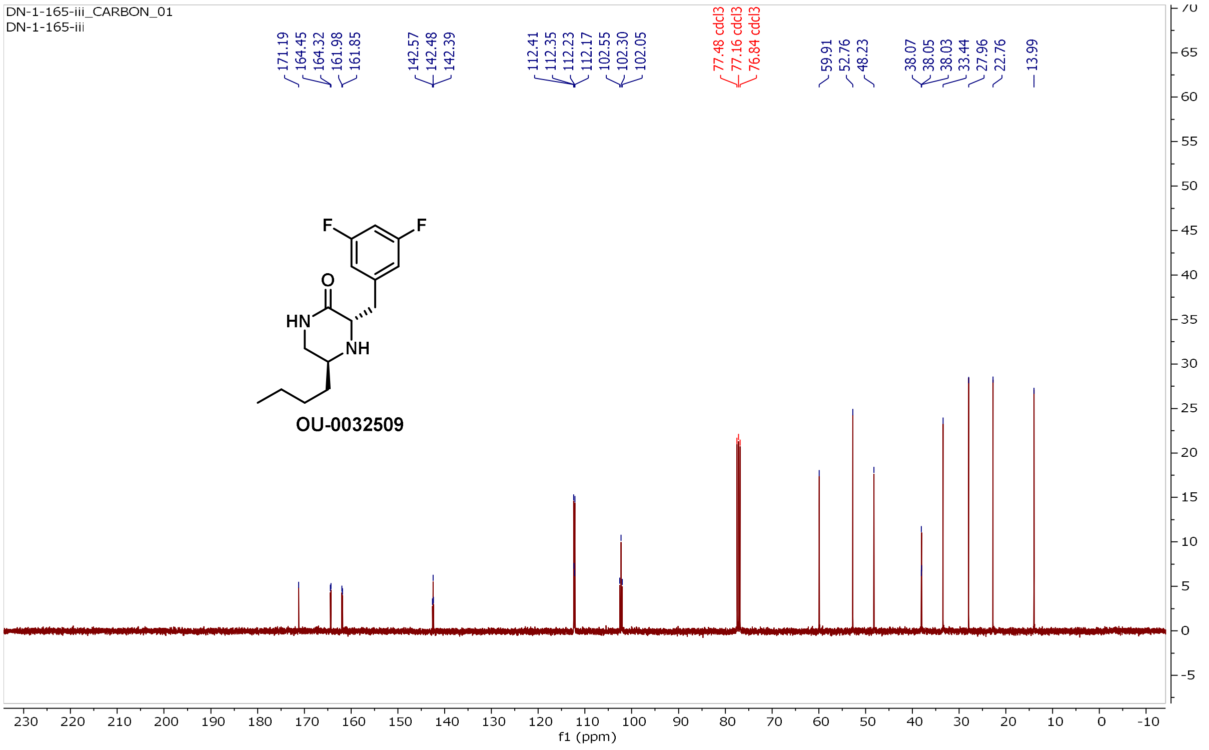
DN-1-165-iii_CARBON_01
DN-1-165-iii
171.19 164.45 164.32 161.98 161.85 142.57 142.48 142.39 112.41 112.35 112.23 112.17 102.55 102.30 102.05 77.48 cdcl3 77.16 cdcl3 76.84 cdcl3 59.91 52.76 48.23 38.07 38.05 38.03 33.44 27.96 22.76 13.99
OU-0032509
f1 (ppm)

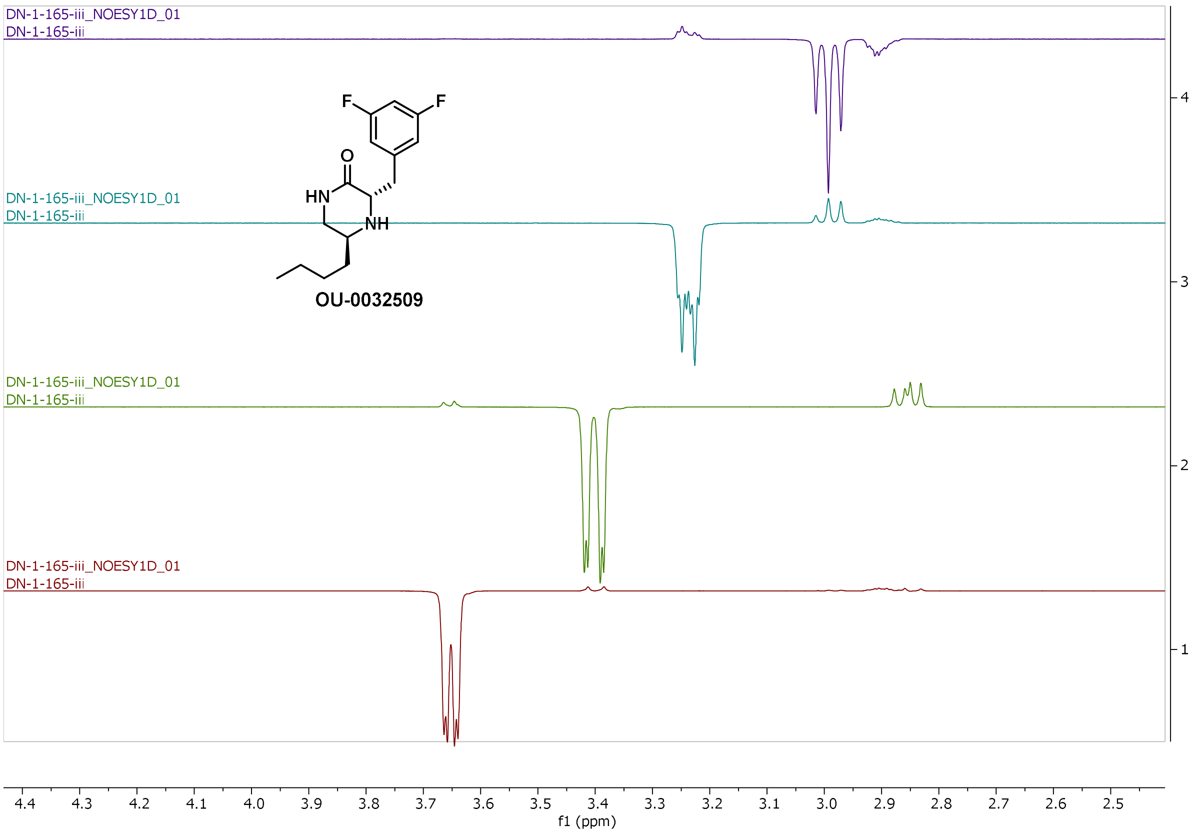
DN-1-165-iii_NOESY1D_01
DN-1-165-iii
DN-1-165-iii_NOESY1D_01
DN-1-165-iii
DN-1-165-iii_NOESY1D_01
DN-1-165-iii
DN-1-165-iii_NOESY1D_01
DN-1-165-iii
OU-0032509
f1 (ppm)

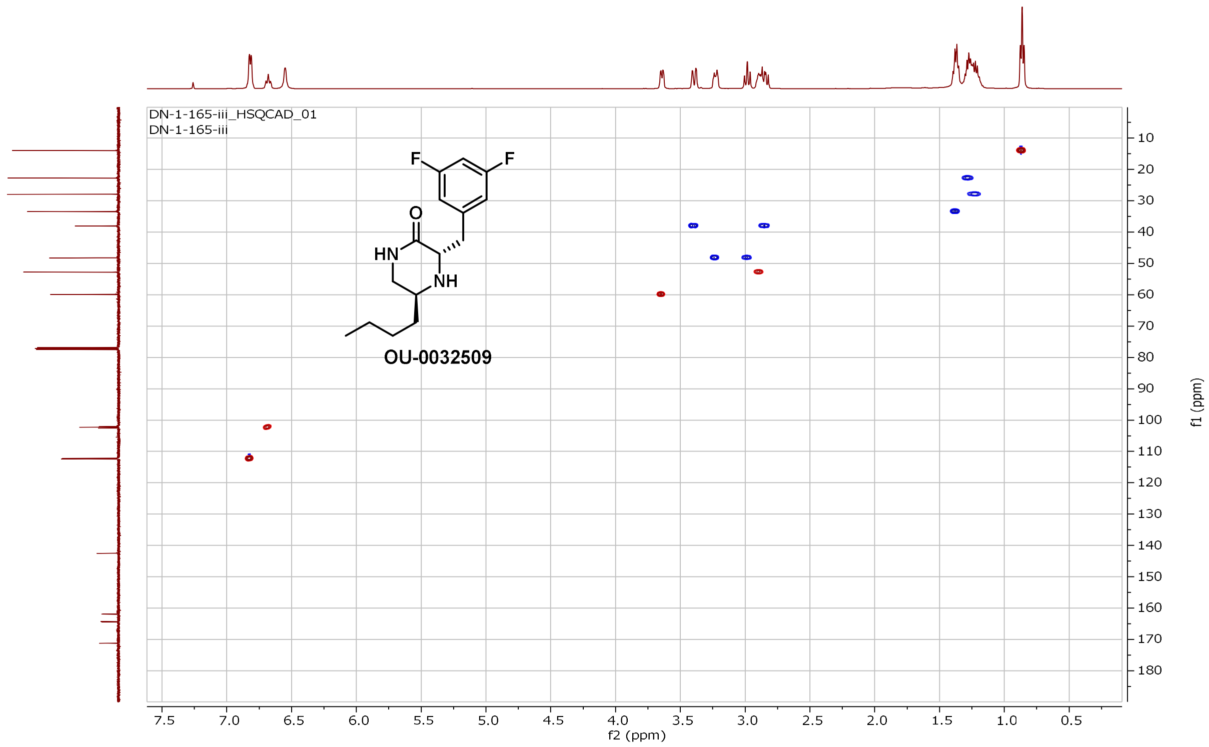
DN-1-165-iii_HSQCAD_01
DN-1-165-iii
OU-0032509
f2 (ppm)
f1 (ppm)

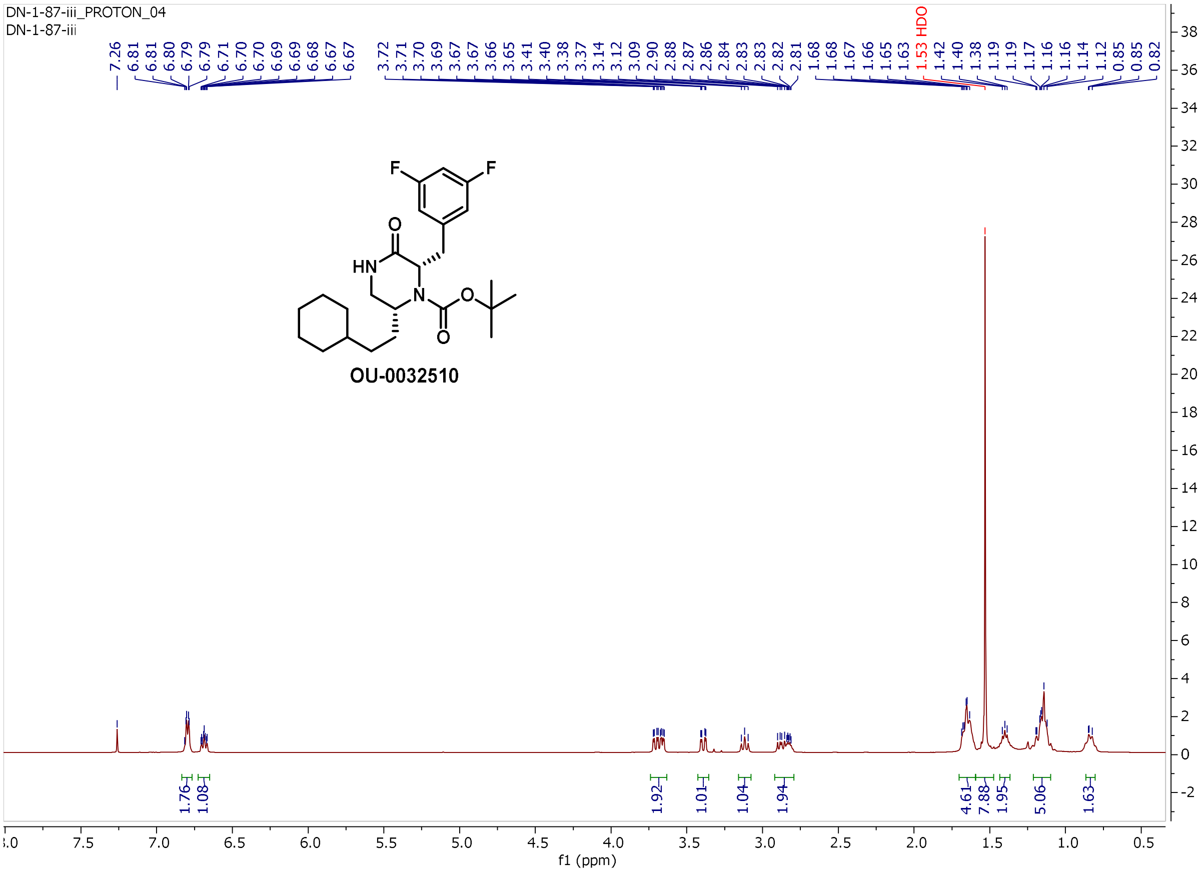
DN-1-87-iii_PROTON_04
DN-1-87-iii
OU-0032510
1.53 HDO
f1 (ppm)

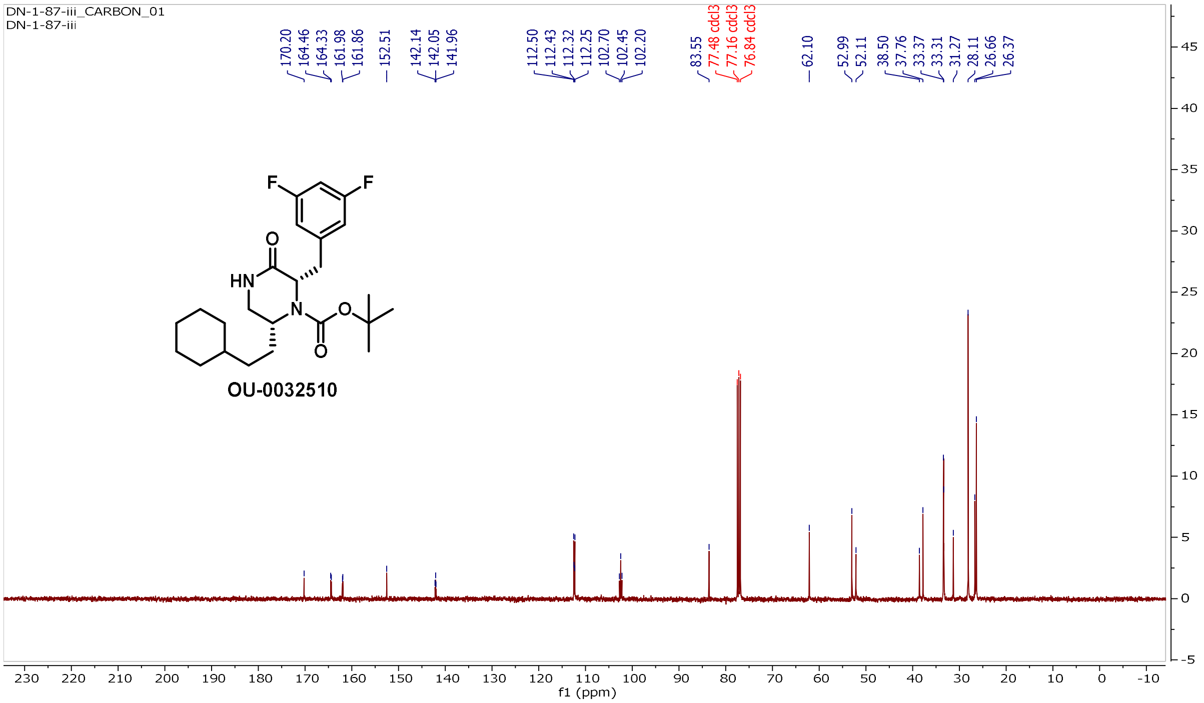
DN-1-87-iii_CARBON_01
DN-1-87-iii
OU-0032510
77.48 cdcl3
77.16 cdcl3
76.84 cdcl3
f1 (ppm)

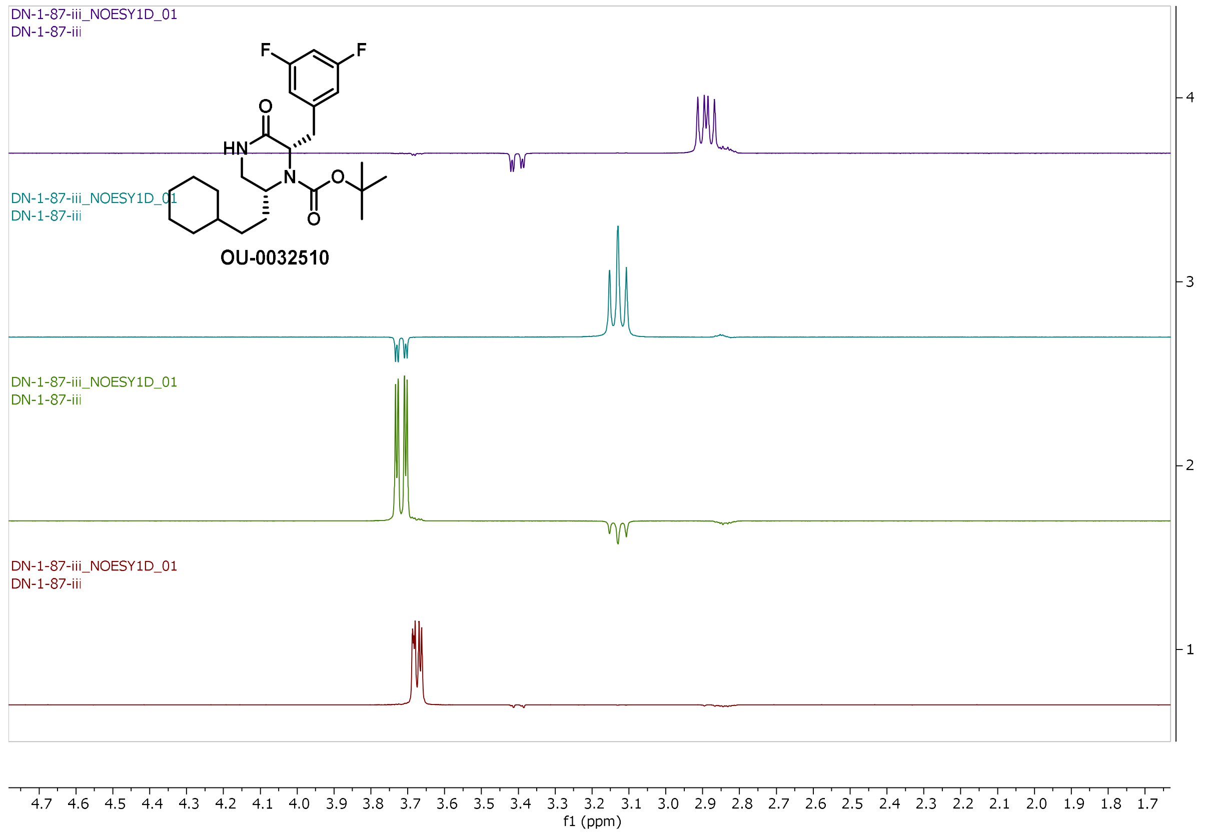
DN-1-87-iii_NOESY1D_01
DN-1-87-iii
OU-0032510
DN-1-87-iii_NOESY1D_01
DN-1-87-iii
DN-1-87-iii_NOESY1D_01
DN-1-87-iii
DN-1-87-iii_NOESY1D_01
DN-1-87-iii
f1 (ppm)

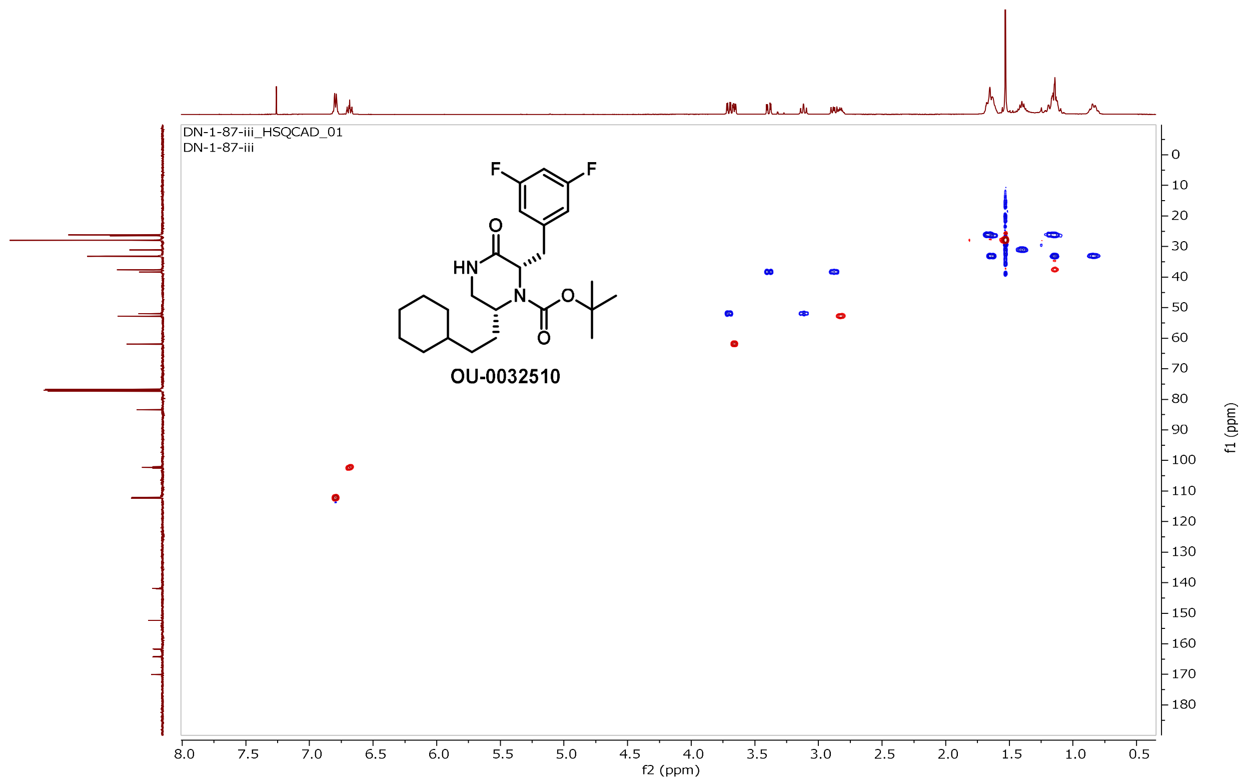
DN-1-87-iii_HSQCAD_01
DN-1-87-iii
OU-0032510
f2 (ppm)
f1 (ppm)

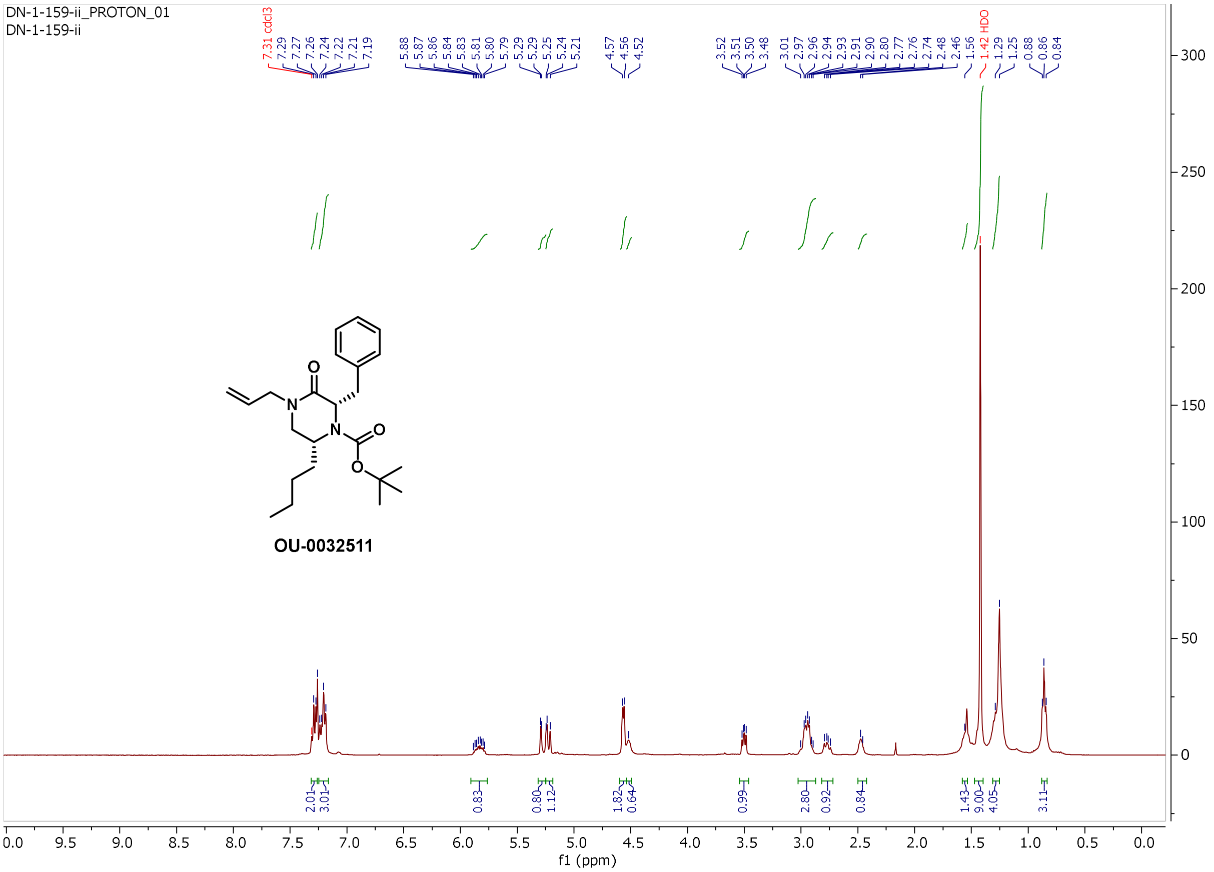
DN-1-159-ii_PROTON_01
DN-1-159-ii
7.31 cdcl3
7.29
7.27
7.26
7.24
7.22
7.21
7.19
5.88
5.87
5.86
5.84
5.83
5.81
5.80
5.79
5.29
5.29
5.25
5.24
5.21
4.57
4.56
4.52
3.52
3.51
3.50
3.48
3.01
2.97
2.96
2.94
2.93
2.91
2.90
2.80
2.77
2.76
2.74
2.48
2.46
1.56
1.42 HDO
1.29
1.25
0.88
0.86
0.84
OU-0032511
2.01
3.01
0.83
0.80
1.12
1.82
0.64
0.99
2.80
0.92
0.84
1.43
9.00
4.05
3.11
f1 (ppm)

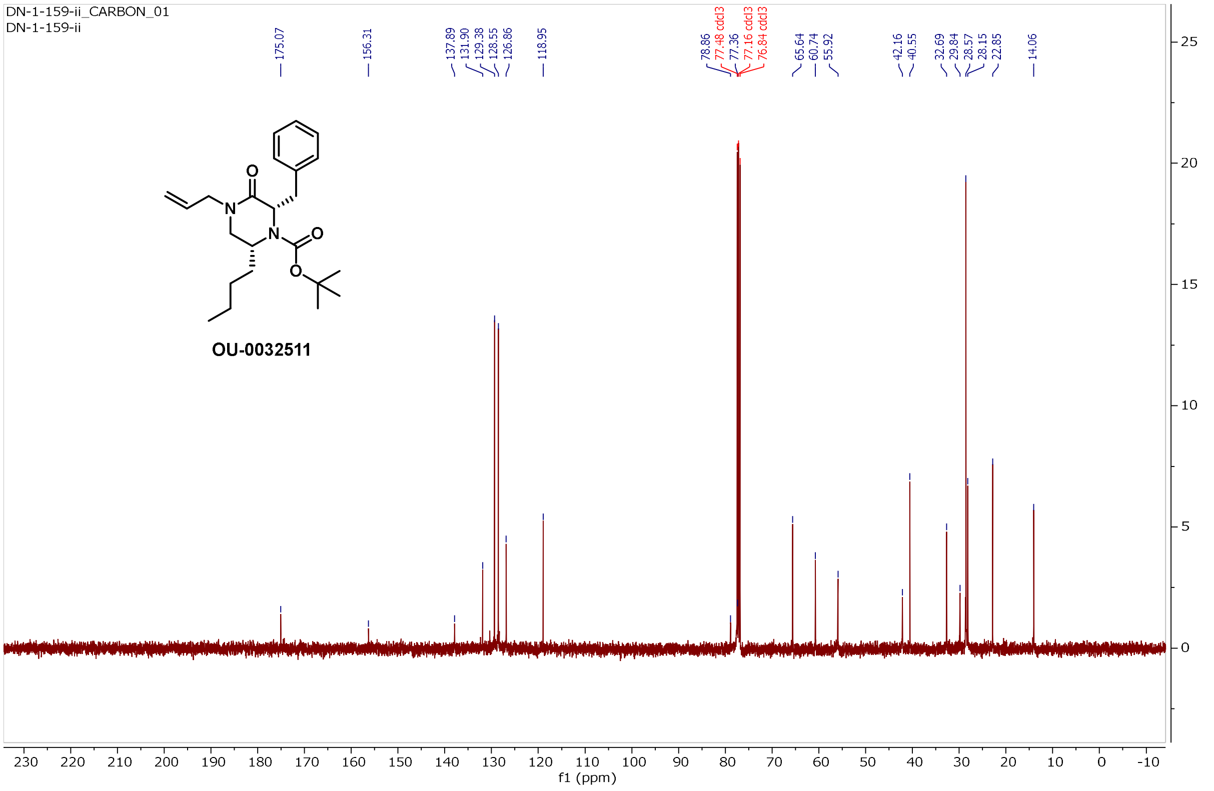
DN-1-159-ii_CARBON_01
DN-1-159-ii
175.07
156.31
137.89
131.90
129.38
128.55
126.86
118.95
78.86
77.48 cdcl3
77.36
77.16 cdcl3
76.84 cdcl3
65.64
60.74
55.92
42.16
40.55
32.69
29.84
28.57
28.15
22.85
14.06
OU-0032511
f1 (ppm)

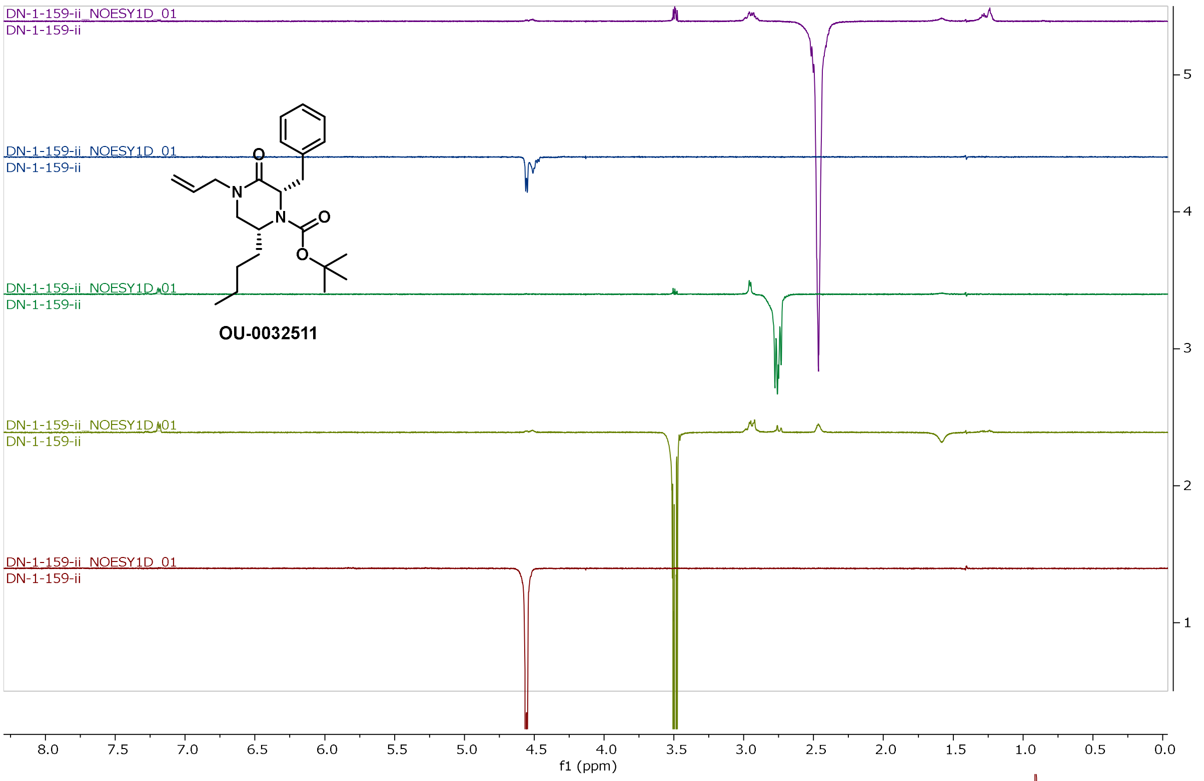
DN-1-159-ii_NOESY1D_01
DN-1-159-ii
OU-0032511
f1 (ppm)

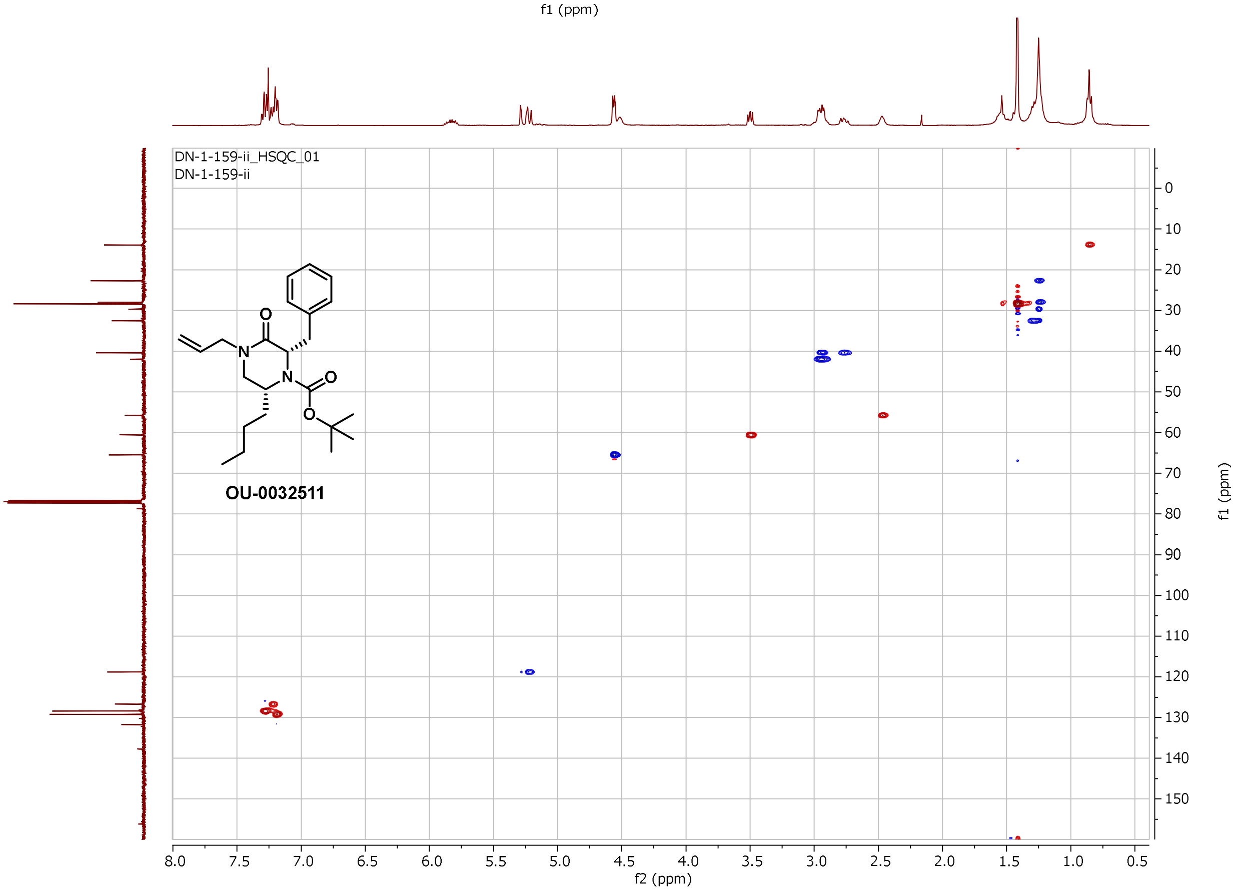
DN-1-159-ii_HSQC_01
DN-1-159-ii
OU-0032511
f2 (ppm)
f1 (ppm)

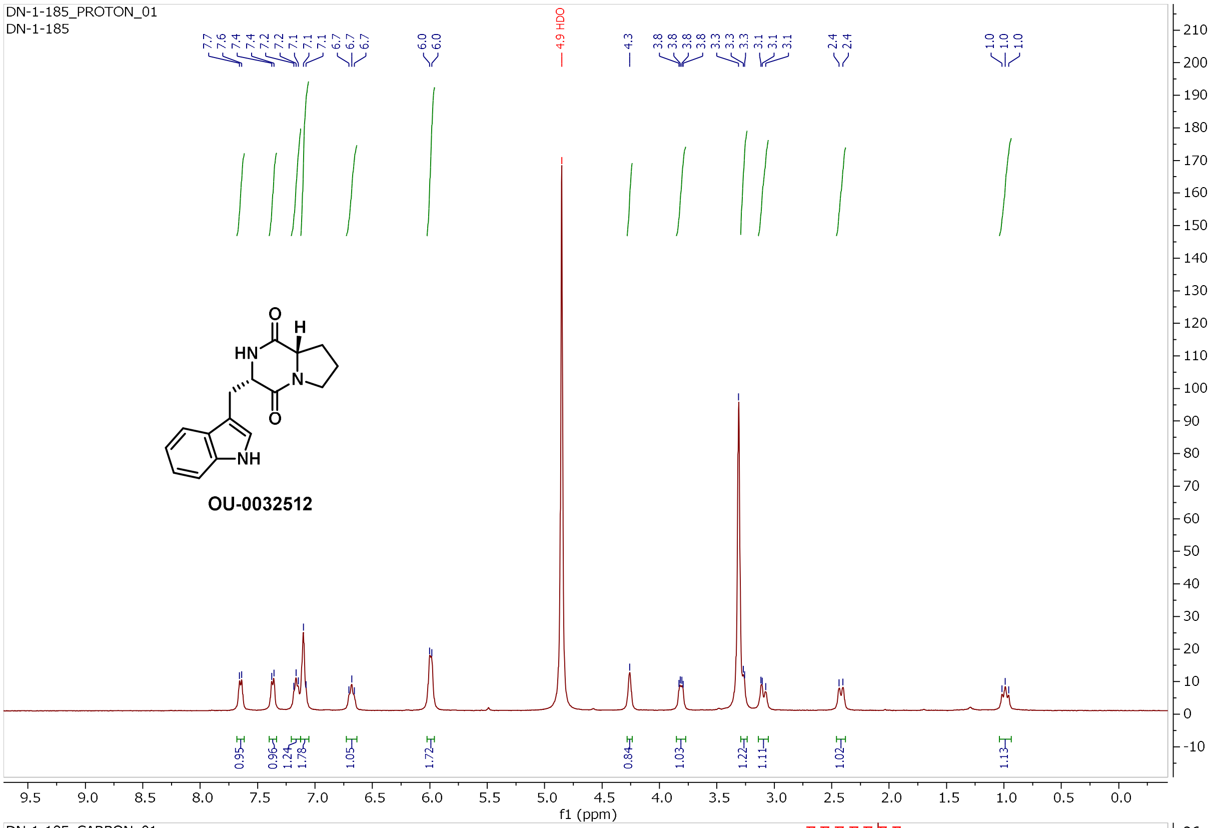
DN-1-185_PROTON_01
DN-1-185
7.7 7.6 7.4 7.4 7.2 7.2 7.1 7.1 7.1 6.7 6.7 6.7
6.0 6.0
4.9 HDO
4.3
3.8 3.8 3.8 3.8 3.3 3.3 3.3 3.1 3.1 3.1
2.4 2.4
1.0 1.0 1.0
0.95 0.96 1.24 1.78 1.05 1.72 0.84 1.03 1.22 1.11 1.02 1.13
OU-0032512
f1 (ppm)

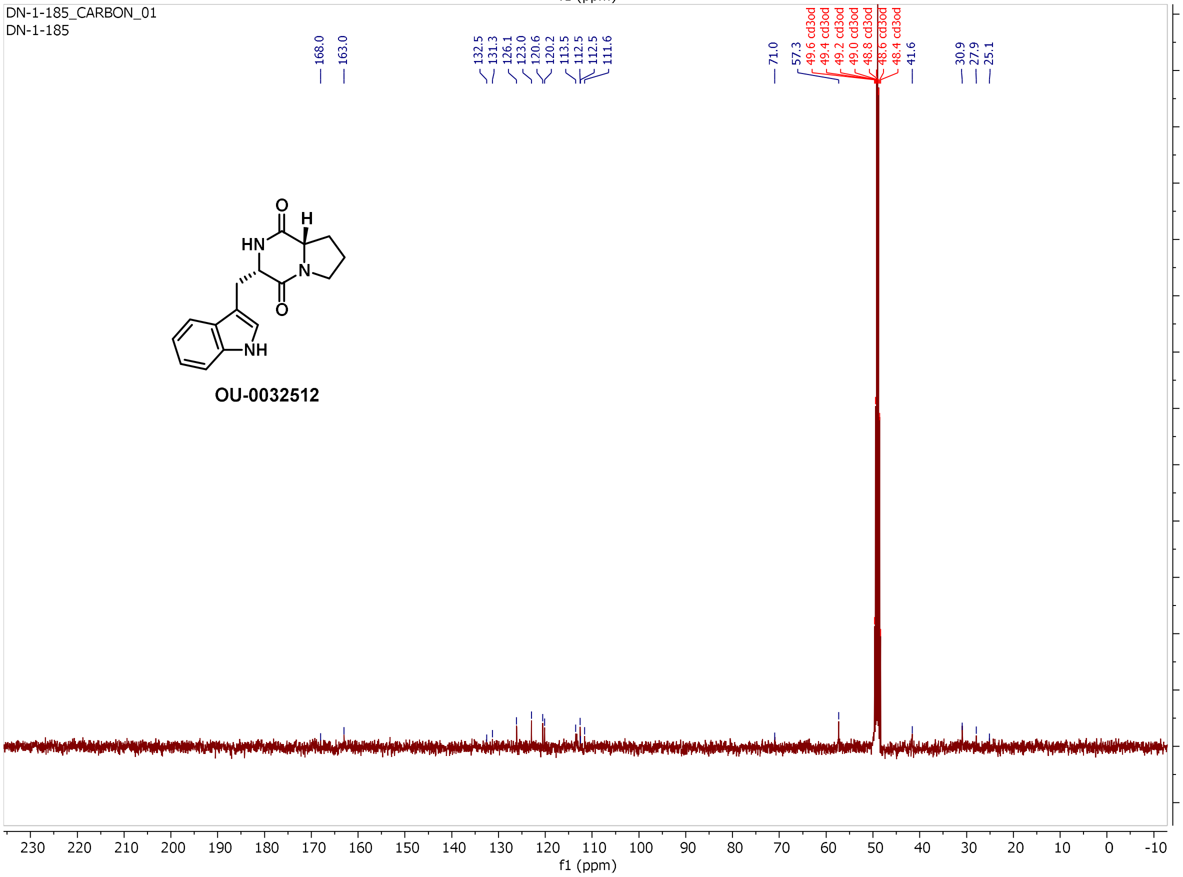
DN-1-185_CARBON_01
DN-1-185
168.0
163.0
132.5 131.3 126.1 123.0 120.6 120.2 113.5 112.5 112.5 111.6
71.0
57.3
49.6 cd3od 49.4 cd3od 49.2 cd3od 49.0 cd3od 48.8 cd3od 48.6 cd3od 48.4 cd3od
41.6
30.9 27.9 25.1
OU-0032512
f1 (ppm)
